# Mental health consequences of urban air pollution: prospective population-based longitudinal survey

**DOI:** 10.1007/s00127-020-01966-x

**Published:** 2020-10-24

**Authors:** Ioannis Bakolis, Ryan Hammoud, Robert Stewart, Sean Beevers, David Dajnak, Shirlee MacCrimmon, Matthew Broadbent, Megan Pritchard, Narushige Shiode, Daniela Fecht, John Gulliver, Matthew Hotopf, Stephani L. Hatch, Ian S. Mudway

**Affiliations:** 1 Health Services and Population Research Department, Centre for Implementation Science, Institute of Psychiatry, Psychology and Neuroscience, King’s College London, London, UK; 2 Department of Biostatistics and Health Informatics, Institute of Psychiatry, Psychology and Neuroscience, King’s College London, London, UK; 3 Department of Psychosis Studies, Institute of Psychiatry, Psychology and Neuroscience King’s College London, King’s College London, London, UK; 4 Department of Psychological Medicine, Institute of Psychiatry, Psychology and Neuroscience, King’s College London, London, UK; 5 NIHR Biomedical Research Centre for Mental Health at the South London and Maudsley NHS Foundation Trust, King’s College London, London, UK, London, UK; 6 MRC Centre for Environment and Health, School of Public Health, Environmental Research Group, Imperial College London, London, UK; 7 Department of Geography, King’s College London, London, UK; 8 MRC Centre for Environment and Health, School of Public Health, Imperial College London, London, UK; 9 Centre for Environmental Health and Sustainability, School of Geography, Geology and the Environment, University of Leicester, Leicester, UK; 10 National Institute for Health Research, Health Protection Research Unit on Environmental Exposures and Health, Imperial College London, London, UK

**Keywords:** Mixed models, Air quality, Common mental disorders, Psychotic experiences, Urban health

## Abstract

**Purpose:**

The World Health Organisation (WHO) recently ranked air pollution as the major environmental cause of premature death. However, the significant potential health and societal costs of poor mental health in relation to air quality are not represented in the WHO report due to limited evidence. We aimed to test the hypothesis that long-term exposure to air pollution is associated with poor mental health.

**Methods:**

A prospective longitudinal population-based mental health survey was conducted of 1698 adults living in 1075 households in South East London, from 2008 to 2013. High-resolution quarterly average air pollution concentrations of nitrogen dioxide (NO_2_) and oxides (NO_*x*_), ozone (O_3_), particulate matter with an aerodynamic diameter < 10 μm (PM_10_) and < 2.5 μm (PM_2.5_) were linked to the home addresses of the study participants. Associations with mental health were analysed with the use of multilevel generalised linear models, after adjusting for large number of confounders, including the individuals’ socioeconomic position and exposure to road-traffic noise.

**Results:**

We found robust evidence for interquartile range increases in PM_2.5_, NO_*x*_ and NO_2_ to be associated with 18–39% increased odds of common mental disorders, 19–30% increased odds of poor physical symptoms and 33% of psychotic experiences only for PM_10_. These longitudinal associations were more pronounced in the subset of non-movers for NO_2_ and NO_*x*_.

**Conclusions:**

The findings suggest that traffic-related air pollution is adversely affecting mental health. Whilst causation cannot be proved, this work suggests substantial morbidity from mental disorders could be avoided with improved air quality.

**Electronic supplementary material:**

The online version of this article (10.1007/s00127-020-01966-x) contains supplementary material, which is available to authorized users.

## Introduction

The Organization for Economic Co-operation and Development (OECD) [1] and World Health Organisation (WHO) [2] rank air pollution as the major environmental cause of premature death and have concluded that by reducing air pollution levels, countries can alleviate the burden of disease by a net benefit of US$ 135,371 million [3]. These estimates are based on the established associations between short- and long-term air pollution exposures and adverse cardiopulmonary morbidity and mortality, but there is now increasing evidence suggesting impacts on neurological endpoints, with an increased focus on pollutants derived from transport sources [4–10]. Observational studies conducted globally have now linked traffic derived air pollution exposures with increased risk of dementia [11], autism spectrum disorders [5], psychotic disorders [6, 7, 9, 10], schizophrenia [12], depression [13], anxiety [14] and cognitive impairment [8] and potential causal pathways have been suggested [15–19]. Specifically, a recent systematic review presented biologically plausible effects of traffic related pollution on cognition with the use of neuroimaging data [20]. These neurological impacts imply significant additional economic and societal costs not currently represented in the WHO [2] and OECD [1] assessments and must be viewed against the reality that the majority of world’s urban populations still breathes air failing to meet the health-based WHO Air Quality Guidelines, especially for PM_2.5_ [21].

Findings from population-based studies of mental health are often limited by: (1) the simplicity of brief screening instruments or proxy measures (e.g. prescription of medication) [7]; (2) over-simplified estimates and surrogates of air pollution measures (e.g. proximity to major roads) [4] or air pollution indices that lack sufficient resolution to capture exposures that vary dramatically over fine spatial scales [9]; (3) cross-sectional designs which fail to measure cumulative exposures and reduce the possibility of reverse causation [10]; (4) failure to measure longitudinal exposures to a range of air pollutants from multiple sources [12] and (5) inadequate control of potential confounders, especially in relation to indices of urbanisation and deprivation (e.g. communities of low socioeconomic status tend to live close to heavy traffic) [8, 22]. Thus, rigorous methodology to confirm the current evidence base is needed [23].

Against this background, we aimed to address the gaps within the existing literature and examine, within a large urban population, the hypothesis that long-term residential exposure to urban air pollution, in an inner-city London area of high-traffic flows is associated with increased risk of common mental disorders, psychotic experiences and symptoms indicative of mental distress, after controlling for large number of confounders, including individuals’ socioeconomic position and exposure to urban noise.

## Methods

### Study area and population

**Fig. 1:**
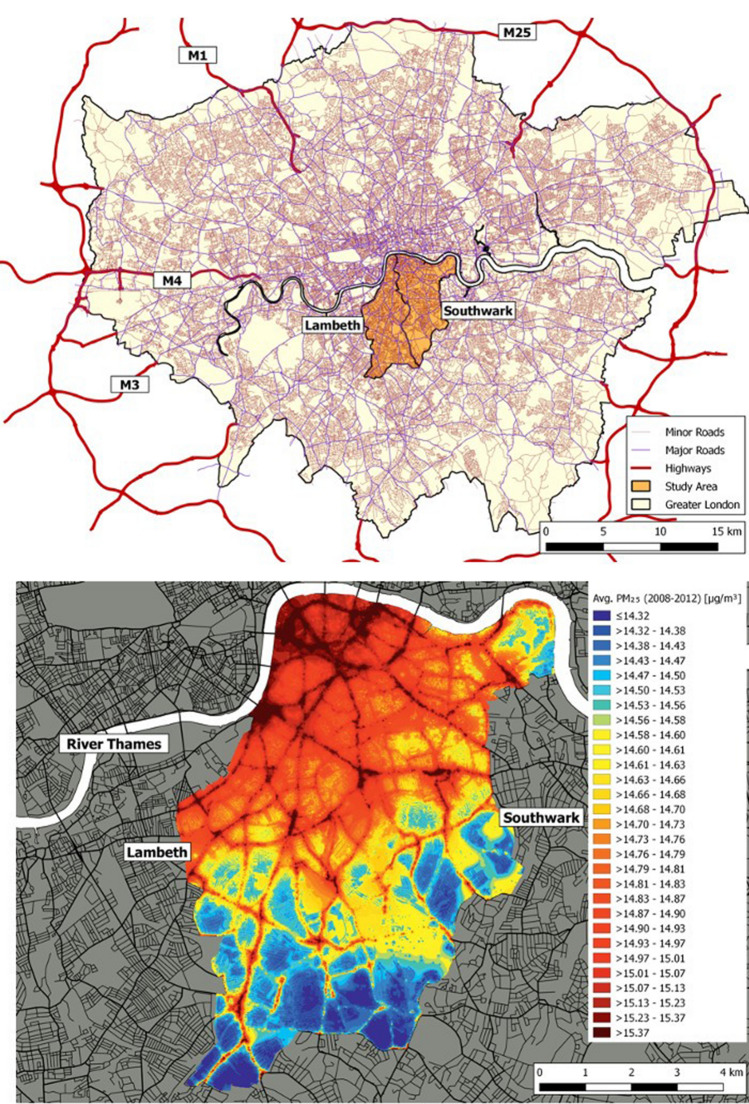
The study area within the London domain is illustrated in the top panel, with the spatial distribution of PM_2.5_ being illustrated in the lower panel, based on the average exposures across 2008–2012 at a resolution of 20 x 20 metre grid points

The South East London Community Health (SELCoH) study is a UK psychiatric and physical morbidity survey of 1698 adults aged 16 years and over residing in 1075 randomly selected households in the boroughs of Southwark and Lambeth between 2008 and 2010, which comprise a total population of 638,200 (mid-2017 estimates) (Fig. 1 and Figure S1). Following the baseline survey (SELCoH 1), 1596 (94%) participants agreed to be re-contacted from 2011 to 2013 and 1052 (73% response rate) participants were re-interviewed (SELCoH 2). The demographic and socioeconomic profiles of the overall sample was similar to the 2011 UK Census demographic and socioeconomic indicators for the catchment area. Details of ethics, study design, geocoding information, sampling techniques, participants, procedures and measures have been published elsewhere [24, 25].

### Measures

At all SELCoH examinations (SELCoH 1 and 2), common mental disorder (CMD) was assessed by the Revised Clinical Interview Schedule (CIS-R), a structured interview administered by trained staff that asks about 14 symptom domains (e.g. fatigue, sleep problems, irritability) [26]. The 14 sub-scale scores are summed to create an overall CIS-R psychological morbidity total score. A conventional CIS-R total score of 12 or more is used to indicate the presence of a CMD [26]. Physical symptoms were measured using the Patient Health Questionnaire subscale (PHQ-15). PHQ-15 screens for 15 somatic symptoms that account for more than 90% of the physical complaints reported in the outpatient setting and has been strongly associated with mental disorders and extensively used in psychiatric research [27]. A total score was acquired by summing all the items in the questionnaire, which was further categorised (0–4, Minimal; 5–9: Low; 10–14; Medium; 15–30: High), due to the skewness of the distribution of the total score. Higher scores indicated more severe symptoms. Fair or poor general health was indicated by a self-rated general health question from the 12-item Short Form (SF-12) questionnaire [28]. We assessed subclinical psychotic experiences using the Psychosis Screening Questionnaire (PSQ) [29], but only within the SELCoH 1 survey, as the questionnaire was not administered in SELCoH 2. Following previous studies that looked at psychotic experiences alone, we excluded question domains related to hypomania and defined psychotic experience as any positive response to secondary questions from the remaining domains [30].

### Long-term and short-term air pollution exposure attributions

We estimated high-resolution (20 × 20 m grid points) exposures at the residential address of the participants from quarterly and annual (2008–2012) nitrogen oxides (NO_*x*_), NO_2_, particulate matter with a diameter of less than 10 µm (PM_10_) and less than 2·5 µm (PM_2.5_) maps of London with the use of the KCL urban model, based on the ADMS dispersion model v4 and road source model v2.3 (Cambridge Environmental Research Consultants), measured hourly meteorological data, empirically derived NO–NO_2_–O_3_ and PM relationships and emissions from the London Atmospheric Emissions Inventory. Sources within the KCLurban model include: road transport (exhaust and non-exhaust), large regulated industrial processes, small regulated industrial processes, large boiler plant, gas heating (domestic and industrial–commercial), oil combustion sources (domestic and commercial), coal combustion sources (domestic and commercial), agricultural and natural sources, rail, ships, airports and others (sewage plant etc.).

Exposure data were outputted as quarterly average concentrations of nitrogen dioxide (NO_2_), nitrogen oxides (NO_*x*_), ozone (O_3_), particulate matter with diameter < 10 μm (PM_10_) and particulate matter with an aerodynamic diameter of < 2.5 mm (PM_2.5_) at the residential address level for the study participants. All pollution exposure estimates were based on the quarterly average estimates at residential address level using the bilinear interpolation method using the 4 points (20 m resolution) around each address point. A comprehensive description of this model, along with information on validation against measurements and its performance against other urban dispersion models has been published previously [31]. Four-year average exposure maps for PM_2.5_ (Fig. 1) and NO_2_ (eFigure S1: Supplementary Online Content) are illustrated for the study area.

### Confounders

The following variables were treated as confounders at the individual level: age, sex, ethnicity, smoking status, latent classes of socioeconomic status, frequency of drinking, physical activity, other chronic conditions and previous mental illness, seasonality and noise from road traffic; and at the neighbourhood level: neighbourhood levels of deprivation, perceived neighbourhood disorder. A continuous age indicator in years was recorded. Self-reported ethnicity indicated identification with one of the following groups: White British, Black Caribbean, Black African, Asian and Other ethnicity. Smoking status was based on four categories—never smoked; current smoker; ex-smoker and sporadic smoker. Latent classes of socioeconomic status (SES) were fitted as categorical indicators of professional homeowners, professional renters, skilled renters, students renters, economically inactive renters, economically inactive homeowners [32]^.^ Frequency of drinking was measured from the first item of the Alcohol Use Disorders Identification Test (AUDIT) [33] “How often do you have a drink containing alcohol?”. Physical activity was derived from a yes and no answer to the question “In the last 4 weeks, outside of work, have you taken part in any sports or vigorous activities or done any exercises (e.g. jogging, bike riding)?”. Participants asked to report any long-standing conditions in relation to asthma, chronic bronchitis, diabetes, high blood pressure, cancer, stroke and previous mental illness. Road-traffic noise was included as road traffic noise levels (dB) modelled to residential postcode centroid using the Traffic Noise Exposure (TRANEX) model [34]. As the temporal variability in noise over the study period was found to be negligible, we modelled noise for one midpoint year (2010) and applied these values to other years for the same address locations across the duration of the study. We considered daytime noise, expressed as annual average A-weighted sound pressure *L*_Aeq,16 h_ (07:00–22:59); night-time noise *L*_Night_ (23:00–06:59) and a day–evening–night noise *L*_den_ (composite *L*_day_, *L*_eve_ with a 5 dB (A) penalty for *L*_eve_ and 10 dB (A) penalty for *L*_night_). As all noise metrics were highly correlated (*ρ* ≥ 0.992) so we therefore use *L*_den_. All noise metrics were positively skewed, and were categorised (*L*_den_: < 60 dB (reference), 60 to < 65 dB and ≥ 65 dB; *L*_Aeq,16 h_: < 55 dB (reference), 55 to < 60 dB, 60 to < 65 dB and ≥ 65 dB; and *L*_night_: < 50 dB (reference), 50 to < 55 dB, 55 to < 60 dB, 60 to < 65 dB and ≥ 65 dB). In order to model long-term patterns, we adjust our models for the calendar years. Perceived neighbourhood disorder was determined from four questions: “Thinking of the area you live in, how much of a problem is each of the following?” asked regarding (1) vandalism/graffiti, (2) crime, (3) safety and (4) rubbish/litter. Responses were scored on a Likert scale as ‘Not a problem’ (0), ‘Minor’ (1), ‘Somewhat serious’ (2) and ‘Very serious’ (3). Total score when all four questions were combined was not normally distributed and so a binary variable was created by splitting the highest rating given on any question into none/minor (low perceived disorder) and somewhat/very serious (high perceived disorder). The UK Official National Statistics Index of multiple deprivation (IMD) 2010 was used to define neighbourhood levels of deprivation. IMD is the government’s official measure of deprivation at the small area level and scores are published for every LSOA in England [35]. The IMD 2010 is based on the data from 2008 for 38 indicators grouped into seven domains and is designed to capture multiple aspects of deprivation. Total IMD contains a health sub-domain which includes measures that aim at estimating local rates of mental disorder, so for this analysis the income and crime subdomains were used on their own as well as overall IMD rank. Although the boroughs of Lambeth and Southwark have areas of low deprivation compared to England as a whole, the majority of both boroughs are more deprived than the national average.

### Statistical analyses

Data analyses were performed using STATA 14.1. Descriptive analyses were weighted for non-response within households. Air pollution exposures (NO_2_, NO_*x*_, O_3_, PM_10_ and PM_2.5_) were analysed as continuous measures, rescaled to both interquartile (IQR) increments and increments specific to the quartile distribution of each air pollutant. Where multiple air pollutants are examined it is a common approach to rescale to the IQR, in order to calculate effect estimates for comparable increases across the different pollutants (which may have very different absolute concentration ranges). Longitudinal associations of air pollutant exposures with CMD (CIS-R), physical symptoms (PHQ-15) and self-rated general health (SF-12) were explored with the use of SELCoH 1 and SELCoH 2. In addition, we restricted our analyses for participants who remained at the same address between SELCoHs 1 and 2. Three-level random intercept logistic and ordinal regression models were used to account for the hierarchical structure of the data, considering observations from baseline (SELCoH 1) and follow up survey (SELCoH 2), individuals at level 2 and households at level 3. Since psychotic experiences were recorded only in SELCoH 1 (and not in SELCoH 2) we explored cross-sectional associations with air pollution metrics with the use of two-level random intercept logistic models considering observations from individuals at level 1 and households at level 2. Initially, all models were fitted separately for each outcome and air pollutant (Model 1-single air pollutant). This was followed by adjustment for age, sex, latent classes of socioeconomic status, smoking status and ethnicity (Model 2-single air pollutant); further adjusted for frequency of drinking and physical activity (Model 3-single air pollutant); further adjusted for *L*_den_ (Model 4-joint air pollutant day–evening-time noise). As a sensitivity analysis we further adjusted separately for (1) each air pollutant with each other; (2) *L*_Night_ and *L*_Aeq,16 h_ (instead of *L*_den_); (3) seasonality (4) neighbourhood levels of deprivation (5) individual’s perceived neighbourhood disorder and (6) previous chronic conditions such as long-standing illness, asthma, chronic bronchitis, diabetes, high blood pressure, cancer, stroke and previous mental illness. We further explored associations between each air pollutant and depression and anxiety scores derived as separate symptom groups from the CIS-R. Effect modification of the association between air pollutants and mental and physical health by latent classes of socioeconomic status was assessed with the inclusion of an interaction term in the above-mentioned models. We also considered annual average concentrations of NO_2_, NO_*x*_, O_3_, PM_10_ and PM_2.5_ in our models—instead of quarterly average concentrations within each year and quarter of the study. Probability weights were included in all the mixed models and took account for non-response within households and attrition between SELCoHs 1 and 2. Finally, we repeated our statistical analyses using the STATA routine ice, an implementation in STATA of the multiple imputations using chained equations (MICE) and compared our results with the original analysis under the missing at random (MAR) assumption [36]. All multilevel models were run with the *gllamm* command [37].

## Results

Longitudinal analyses were conducted in 1052 participants who participated in both surveys (SELCoH 1 and 2). Cross-sectional analyses for psychotic experiences measured only in SELCoH 1 were conducted on 1655 individuals. Descriptive statistics are presented in Table 1.

**Table 1:** Characteristics of the study population and distribution of mental health outcomes, exposures and confounders within the two waves of the South East London Community Survey (SELCoH 1 and 2)

Survey	SELCoH 1		SELCoH 2	
	Number (%) unless otherwise stated	*n*	Number (%) unless otherwise stated	*n*
Age	Mean: 40; SD: 16.9	1698	Mean: 43; SD: 16.5	1052
Gender				
Male	737 (47.6)	1698	437 (47.5)	1052
Female	961 (52.4)		615 (52.5)	
Latent classes of socioeconomic status				
Professional, homeowners	470 (27.8)	1698	351 (32.6)	1052
Professional, renters	112 (6.9)		43 (4.7)	
Skilled, renters	351 (20.7)		244 (22.6)	
Students, renters	230 (14.5)		103 (12.4)	
Economically inactive, renters	407 (23.0)		213 (19.4)	
Economically inactive, homeowners	128 (7.1)		98 (8.1)	
Smoking status				
Never smoked	514 (30.4)	1685	426 (40.4)	1052
Current smoker	423 (25.6)		225 (22.6)	
Ex-smoker	450 (26.0)		350 (31.4)	
Sporadic smoker	298 (18.0)		51 (5.2)	
Frequency of alcoholic drink				
Never	379 (22.0)	1689	212 (18.9)	1052
Monthly or less	377 (22.2)		228 (21.8)	
Two or four times a month	290 (17.4)		183 (18.4))	
Two or three times a week	380 (22.8)		256 (24.9)	
Four or more times a week	263 (15.6)		173 (15.8)	
Physically active				
No	722 (42.0)	1670	408 (41.3	1052
Yes	948 (58.0)		644 (58.6)	
Ethnicity				
White	1051 (61.6)	1698	688 (63.8)	1052
Black Caribbean	143 (8.4)		79 (7.8)	
Black African	234 (14.0)		131 (13.0)	
Asian	63 (3.7)		40 (3.8)	
Other	205 (12.3)		114 (11.4)	1
Perceived neighbourhood disorder				
None/minor	1042 (62.5%)	1666	1042 (61.6%)	1051
Somewhat/very serious	626 (37.6%)		403 (38.3%)	
Index of multiple deprivation	Mean: 30.4; SD: 8.4	1666	Mean: 29.7; SD: 8.5	1051
Air pollutant median concentrations (μg/m^3^)				
NO_2_	Median: 39.6 (IQR: 17.3)		Median: 35.8 (IQR: 19.6)	
NO_*x*_	Median: 67.4 (IQR: 45.6)		Median: 57.0 (IQR: 48.0)	
O_3_	Median: 31.7 (IQR: 21.3)		Median: 35.7 (IQR: 14.9)	
PM_10_	Median: 22.6 (IQR: 3.6)	1698	Median: 18.5 (IQR: 4.6)	937
PM_2.5_	Median: 14.2 (IQR: 3.2)		Median: 13.7 (IQR: 2.6)	
24-h noise metric (*L*_den_) (dB)				
< 60	1196 (70.4)	1698	729 (69.4)	1052
[60, 65)	160 (9.5)		96 (9.1)	
≥ 65	342 (20.1)		227 (21.5)	
Daytime noise metric (*L*_Aeq,16 h_) (dB)				
< 55	329 (19.3)	1698	196 (18.1)	1052
[55, 60)	983 (58.0)		598 (56.5)	
[60, 65)	155 (9.1)		103 (9.8)	
≥ 65	231 (13.6)		155 (14.6)	
Night-time noise metric (*L*_night_) (dB)				
< 50	808 (47.7)	1698	493 (46.9)	1052
[50, 55)	494 (29.2)		143 (28.2)	
[55, 60)	143 (8.3)		93 (8.8)	
[60, 65)	171 (9.9)		112 (10.1)	
≥ 65	82 (4.9)		58 (6.0)	
Revised Clinical Interview Schedule (CIS-R)				
< 12	1296 (77.0)	1692	821 (77.9)	1052
12 +	396 (22.9)		231 (22.1)	
Patient Health Questionnaire (PHQ-15)				
Minimal (0–4)	936 (60.6)	1567	553 (55.9)	1000
Low (5–9)	439 (27.6)		290 (28.7)	
Medium (10–14)	153 (9.4)		121 (11.8)	
High (15–30)	39 (2.3)		36 (3.5)	
12-Item Short Form Health Survey (SF-12)				
Excellent/very good	821 (48.9)	1688	548 (53.4)	1052
Good	571 (34.1)		308 (29.3)	
Fair/poor	296 (17.0)		196 (17.3)	
Psychotic experiences				
No	1382 (81.3)	1686	NA	NA
Yes	304 (18.7)		NA	NA
Long-standing illness	654 (39.2)	1666	487 (46.3)	1051
Asthma	132 (7.9)	1666	100 (9.5)	1051
Chronic bronchitis	8 (0.4)	1666	6 (0.5)	1051
Diabetes	73 (4.3)	1666	57 (5.4)	1051
High blood pressure	150 (9.0)	1666	142 (13.5)	1051
Cancer	23 (1.3)	1666	17 (1.62)	1051
Stroke	13 (0.7)	1666	16 (1.5)	1051
Previous mental illness	100 (6.0)	1666	81 (7.7)	1051

Weighted percentages are presented to account for survey design; frequencies are unweighted and may not add up due to missing values

### Is air pollution associated with CMD and psychotic experiences?

**Fig. 2:**
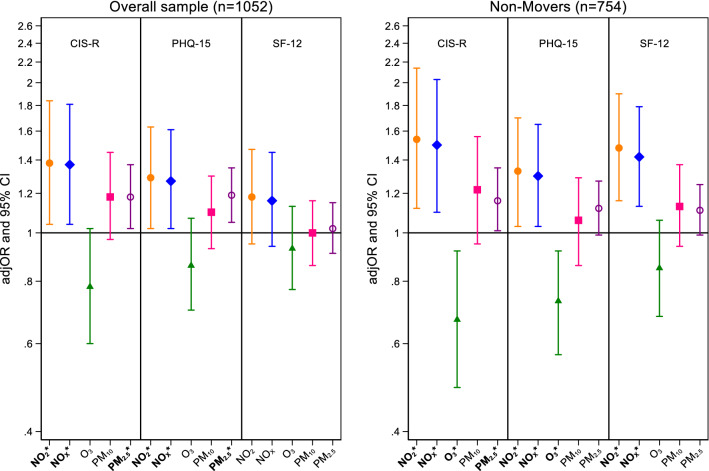
Adjusted odds ratios (adjOR) and their corresponding 95% intervals (CI) represent increase in risk for common mental disorders (CIS-R), physical symptoms (PHQ-15) and self-rated general health (SF-12) per IQR increase in air pollutant (NO_2_, NO_*x*_, O_3_, PM_10_, PM_2.5_) levels (μg/m^3^). All models are adjusted for age, sex, latent classes of SES, smoking status, ethnicity, frequency of drinking, physical activity and *L*_den_

**Fig. 3:**
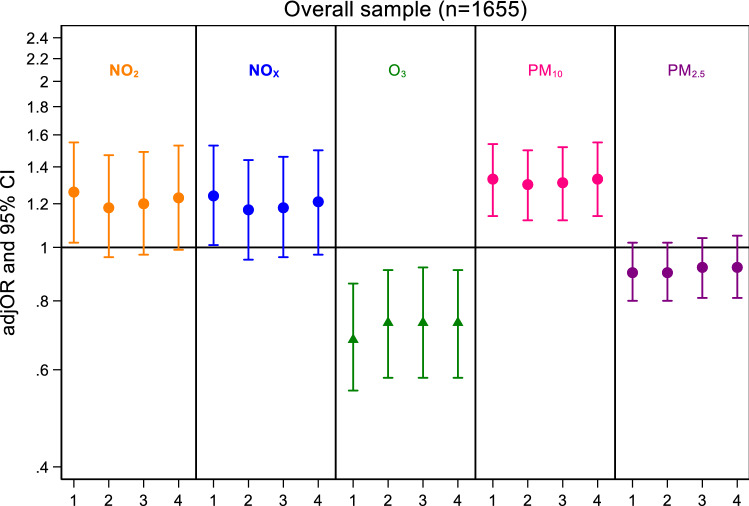
Adjusted odds ratios (adjOR) and their corresponding 95% intervals (CI) represent increase in risk for psychotic experiences per IQR increase in air pollutant (NO_2_, NO_*x*_, O_3_, PM_10_, PM_2.5_) levels (μg/m^3^). Model 1: unadjusted; Model 2: Adjusted for age, sex, latent classes for SES, smoking status, ethnicity; Model 3: Adjusted for age, sex, latent classes for SES, smoking status, ethnicity, frequency of drinking, physical activity ^±±±±^Model 4: for age, sex, latent classes for SES, smoking status, ethnicity, frequency of drinking, physical activity and *L*_den_

The results from univariate longitudinal analyses presented positive associations between NO_2_, NO_*x*_ and PM_2.5_ with CMD (all *p* values < 0.05). The results of the univariate analyses were replicated in the multivariate analyses, when adjusted for age, sex, latent classes of SES, smoking status, ethnicity, frequency of drinking, physical activity and *L*_den_. Here consistent positive longitudinal associations with CMD were seen for NO_2_ (OR 1.39; 95% CI 1.05, 1.85) NO_*x*_ (OR 1.37; 95% CI 1.04, 1.81) and PM_2.5_ (OR 1.18; 95% CI 1.02, 1.37) (Fig. 2 and Table 2). When we restricted our analyses to non-movers, stronger associations were observed for CMD. Specifically, after adjusting for all confounders, the odds ratio for CMD were 1.54 (95% CI 1.12, 2.14) and 1.50 (95% CI 1.10, 2.03), respectively, for NO_2_ and NO_*x*_ (Fig. 2 and eTable S1: Supplementary Online Content). For non-movers, we also observed negative associations between O_3_ and CMD (Fig. 2 and eTable S1: Supplementary Online Content). In addition, our per quartile analysis presented an almost twofold increase in CMD for participants with exposure in the 4th quartile (> 24 μg/m^3^) compared to participants with exposure to the 1st quartile (12.4 μg/m^3^) for PM_2.5_ for the overall sample of SELCoH 1 and 2 (*n* = 1052; OR 1.93; 95% CI 1.22, 3.05) and non-movers (*n* = 754; OR 1.77; 95% CI 1.03, 3.25) (eTable S2 and eTable S3: Supplementary Online Content). PM_10_ and Psychotic experiences extracted from SELCoH 1 showed strong evidence for a cross-sectional association with each air pollutant (OR 1.33; 95% CI 1.14, 1.55; Model 4; Fig. 3).

**Table 2:** Longitudinal associations between air pollutants (NO_2_, NO_*x*_, O_3,_ PM_10,_ PM_2.5_) and common mental disorders (CIS-R), physical symptoms (PHQ-15) and self-rated general health (SF-12) with the use of the SELCoH 1 and 2 surveys

	Model 1^±^	Model 2^±±^	Model 3^±±±^	Model 4^±±±±^
	OR 95% CI	OR 95% CI	OR 95% CI	OR 95% CI
NO_2_				
CIS-R	1.44* 1.07,1.92	1.36* 1.02, 1.82	1.39* 1.05, 1.85	1.39* 1.05, 1.85
PHQ-15	1.33* 1.04, 1.71	1.28* 1.01, 1.62	1.30* 1.03, 1.65	1.30* 1.02, 1.64
SF-12	1.20 0.95,1.52	1.15 0.93,1.44	1.18 0.95,1.47	1.17 0.94,1.46
NO_*x*_				
CIS-R	1.41* 1.06, 1.88	1.35* 1.02, 1.79	1.38* 1.04, 1.82	1.37* 1.04, 1.81
PHQ-15	1.31* 1.03, 1.66	1.26* 1.01, 1.58	1.29* 1.02, 1.62	1.28* 1.02, 1.61
SF-12	1.17 0.93, 1.47	1.13 0.91, 1.40	1.16 0.94, 1.43	1.15 0.93, 1.42
O_3_				
CIS-R	0.77 0.59, 1.01	0.80 0.61, 1.10	0.78 0.60, 1.02	0.78 0.60, 1.02
PHQ-15	0.86 0.69, 1.07	0.89 0.72, 1.10	0.86 0.70, 1.07	0.86 0.70, 1.07
SF-12	0.96 0.78, 1.18	0.97 0.80, 1.18	0.93 0.77, 1.13	0.93 0.77, 1.13
PM_10_				
CIS-R	1.25* 1.01, 1.54	1.19 0.97, 1.46	1.19 0.97, 1.45	1.19 0.97, 1.45
PHQ-15	1.13 0.94, 1.35	1.11 0.93, 1.31	1.11 0.93, 1.31	1.10 0.93, 1.30
SF-12	1.03 0.88, 1.21	1.00 0.86, 1.16	1.00 0.86, 1.16	1.00 0.86, 1.16
PM_2.5_				
CIS-R	1.20* 1.03, 1.41	1.18* 1.01, 1.37	1.18* 1.02, 1.38	1.18* 1.02, 1.37
PHQ-15	1.22** 1.07, 1.39	1.20** 1.06, 1.36	1.19** 1.05, 1.35	1.19** 1.04, 1.35
SF-12	1.07 0.94, 1.22	1.03 0.92, 1.16	1.02 0.91, 1.15	1.02 0.91, 1.15

Odds ratios (OR) and their corresponding 95% intervals (CI) represent increase in risk for mental disorders and physical symptoms per IQR increase in air pollutant levels (μg/m^3^)

*p < 0.05, **p < 0.01 ^±^Model 1: unadjusted ^±±^Model 2: Adjusted for age, sex, latent classes of SES, smoking status, ethnicity ^±±±^Model 3: Adjusted for age, sex, latent classes of SES, smoking status, ethnicity, frequency of drinking, physical activity ^±±±±^Model 4: Adjusted for age, sex, latent classes of SES, smoking status, ethnicity, frequency of drinking, physical activity and *L*_den_

### Is air pollution associated with poor psychical symptoms and self-rated general health?

The results from univariate and multivariate longitudinal analyses showed positive associations for NO_2_, NO_*x*_ and PM_2.5_ with an increased total score for PHQ-15 (all *p* values < 0.05) (Fig. 2 and eTable S2: Supplementary Online Content). Similar odds ratios were observed when we restricted our analyses to non-movers (eTable S1-Supplementary Online Content). For SF-12, none of the air pollutants showed association (Fig. 2 and Table 2), yet, for non-movers only, SF-12 showed a positive association with NO_2_ and NO_*x*_ (all *p* values < 0.05) and negative associations with O_3_ (eTable S2 and eTable S3: Supplementary Online Content).

### Sensitivity analyses

All odds ratio estimates were attenuated when two-air pollutant models were employed—this is due to issues of high multicollinearity (all Pearson correlation coefficients > 0.75 between the air pollutants and the variance inflation factor, an indicator of multicollinearity, which was > 10 for all models; eTable S4). Modifying our model to account for the road-traffic noise separately for *L*_Night_ and *L*_Aeq,16 hr_ (eTable S4) or employing annual average concentrations of air pollutants (instead of quarterly) (eTable S5) made no significant difference in the estimates of odds ratio and 95% confidence intervals of models. Further adjusting separately for other chronic conditions, seasonality, neighbourhood levels of deprivation and perceived neighbourhood disorder also made no significant difference to our results (eTable S6 and eTable 7: Supplementary Online Content). When we derived depression and anxiety scores as separate symptom groups from the CIS-R no evidence of an association were observed with each air pollutant, although direction of odds ratio was on the expected direction (eTable 8-Supplementary Online Content). No evidence of an effect modification of the association was found between each outcome and air pollutant by individuals’ SES (all *p* values > 0.05; eTable S9). In addition, odds ratio estimates did not change substantially when we replicate our analyses with the use of the MICE procedure (eTable S10).

## Discussion

In this study, we addressed the association between air pollution, CMD and psychotic experiences in adults within a high traffic inner city area. We demonstrated consistent longitudinal associations of long-term exposure to air pollutants (NO_2_, NO_*x*_ and PM_2.5_) with mental disorders and physical symptoms indicative of mental distress based on the standardised and validated interviews and questionnaires, after adjusting for a large number of confounders, including individual level socioeconomic status and urban noise. These associations were more pronounced for NO_2_ and NO_*x*_ in the subset of non-movers across the two-survey dates. Our data also support a cross-sectional association between PM_10_ exposure and psychotic experiences. There was no evidence to suggest modification of the observed associations by socioeconomic characteristics.

### Strengths and limitations of this study

The present study investigated a large, representative group of individuals within an inner-city population, reflecting the London’s ethnic diversity and broad spectrum of socioeconomic conditions. The study area consistent of 2 densely populated London boroughs of Lambeth and Southwark (combined population of 638,200), which experience similar levels of annual air pollution compared to London (e.g. median levels of PM_2.5_ are 14.2 μg/m^3^ vs. London 14.4 μg/m^3^ levels [38]). For air pollution exposures, our study had higher spatial [9, 12] and temporal precision than previous work [10, 39], thereby reducing potential exposure misclassification, especially for primary traffic-related air pollutants such as NO_2_. However, some exposure misclassification remains as the participants of the SELCoH study may have had different exposure patterns due to their everyday mobility patterns or being away from their residence (e.g. workplace or transport) for a large percentage of their day or indoor air pollution exposures or exposure modification owing to behaviours (e.g. opening windows) or building characteristics (e.g. bedroom façade). The significance of this exposure misclassification was potentially greatest for pollutants that varying in concentrations markedly with distance from source, such a NO_2_ or where there was marked diurnal and seasonal variations, e.g. O_3_. A novel aspect of our analysis was the ability to study the modelled effects of air pollution on each outcome after adjusting for road-traffic noise, overcoming acknowledged limitations from other studies [7, 12]. We therefore believe that the associations observed are robust and were persistent in multiple sensitivity analyses. In addition, the fact that our study revealed these outcomes with a relatively narrow range of exposures, with the majority of subjects living in high traffic areas, implies that a more pronounced effect may exist between urban and rural populations.

One limitation of the study is that we were unable to consider the association between mental and physical health with shorter term, e.g. daily fluctuations in air pollution. Furthermore, a previous study had shown only moderate correlations between the air pollution and noise models used in this study, thus indicating that noise is not a potential candidate to explain the association between traffic-related air pollution [40]. We used a detailed noise model which follows the UK Calculation of Road Traffic Noise method [34]. The model takes into account noise barriers, including buildings and land cover which accounts to some degree for vegetation. Our noise model is likely to have overestimated and underestimated noise on some minor roads owing to the use of a constant for traffic on minor roads where detailed traffic data were not available. However, in order to reduce potential exposure misclassification, we categorised noise exposure for our analysis, when we further adjusted our models for road traffic noise Consistent with this view, we observed little change in effect size after adjusting for road-traffic noise. The 51.9% household participation rate and the 38.1% attrition rate were low and we were unable to characterise non-respondents’ demographic variables to rule out possible bias owing to non-participation. Nevertheless, samples were representative of the local population on most sociodemographic characteristics [25] and probability weights were estimated to address both household participation and sample attrition in our statistical models. Furthermore, multiple imputations were employed as additional sensitive analysis to handle attrition in our models. A further weakness of this study, which is shared with the majority of studies on this topic, is the lack of air pollution data capturing an accurate picture of lifetime or cumulative exposure Because of central nervous system plasticity during development, children are particularly susceptible to harmful effects of air pollution on neurodevelopment and therefore to long-term cognitive health. Therefore, one would wish to capture early life exposures to provide comprehensive understanding of how changes in individual cognitive trajectories might influence transition to lower IQ, attention deficit hyperactivity disorder (ADHD) and autism spectrum disorder during childhood and psychiatric symptoms in adulthood [41]. Unfortunately, these links have been largely unexplored in the current literature. In our study, the quantification of the impact of air pollution on mental and psychical health has been undertaken through a single pollutant approach, due to the measurement and source complexities and regulatory strategies of air quality management, which have addressed a single pollutant at a time. However, from a modelling perspective effect estimates could be augmented with a multi-pollutant approach [42, 43] as currently they might provide an underestimation of the effect sizes.

### Comparison to other studies

Our single air pollutant model findings are partly consistent with recent studies in adults, which report increased risk of mental disorders with NO_2_ and PM_2.5_. The majority of previous studies have focused on depression and anxiety and presented positive associations with increased concentrations of NO_2_ [39] and PM_2.5_ [13, 14]. Our study is the first to assess the relationship between long-term exposure to air pollution (PM_10_) and psychotic experiences in the UK in adults. A recent UK study has reported similar effect size of long-term exposure to PM_10_ and elevated risk in psychotic experiences in adolescents (OR 1.27; 95% CI 0.98–1.65) [10]. In Sweden, children and adolescents residing in areas with high PM_10_ concentrations were more likely to have a dispensed medication for a psychiatric disorder than those residing in areas with low PM_10_ concentrations [7]. In adults, a summary measure of air quality constructed from a wide range of environmental compounds was associated with increased risk of schizophrenia in the US and Denmark [9]. When a re-analysis of the same cohort was conducted with high-resolution air pollution estimates, inconclusive evidence was observed between PM_10_ and schizophrenia [44]. Short-term acute effects of PM_10_ were also associated with increased risk of psychosis morbidity [45] and hospital admissions for schizophrenia [46] in two studies conducted in China. NO_2_ and NO_*x*_ as the sum of NO_2_ and nitric oxide (NO), are associated with motor vehicle exhausts and are common markers of traffic-related pollution, specifically in cities while PM_10_ and PM_2.5_ are commonly used as a proxy of ambient air pollution [47]. Our results for a negative association of ozone (O_3_) with CMD are contradictory to similar studies published previously on the topic [23]. This is due to the fact that O_3_ has an opposite spatial distribution than e.g. NO_2_ and we would therefore expect a negative association given the positive association seen with NO_2_, indicating that traffic-related air pollutants are driving the observed patterns.

### Potential mechanisms

A broad range of psychiatric conditions have been associated with systemic and CNS inflammation and oxidative stress [15, 48, 49] and neurogenerative pathophysiologic processes [50, 51] following air pollution exposures. Animal studies have further demonstrated that inflammation and oxidative stress may also affect the CNS [15, 52, 53]. In an early seminal study, exploring whether air pollution may be a risk factor of neurogenerative disease, healthy feral dogs chronically exposed to traffic-related pollution showed enhance oxidative, immunological and genetic damage in olfactory bulbs, frontal, cortex and hippocampus [54]. Recent evidence has also demonstrated elevated numbers of combustion derived magnetite nanoparticles in the brains of urban dwellers [17], associated with pathological alterations in neurons, glia and neurovascular units [18] with evidence of enhanced particle numbers in archived brains of dementia [55]. Neuroinflammation and neurotoxicity appears to be important both for depression [56] and psychosis [57] for both short- and long-term effects of air pollution. Furthermore, uncertainties still exist on how inhaled nanoparticles (particulate and ultrafine particles) gain access to the brain and alter brain structure [19, 20, 55, 58]. Particulate matter may enter brain via (1) the lungs, which could induce respiratory tract inflammation and could result in activation of microglia and oxidative, immunological and genetic damage; (2) the bloodstream, crossing the blood–brain barrier; (3) the nasal pathway and the olfactory nerve where nanoparticles travel directly to the brain, producing direct toxic damage to the limbic system and brain degeneration due to oxidative stress [55, 59].

## Conclusions

Our results are consistent with urban air pollution having a significant impact on poor mental health, which cannot be explained by other indices of urbanicity or socioeconomic deprivation, although an underlying mechanistic understanding of causation is still required to substantiate this linkage. We estimate a twofold increase in terms of common mental disorder cases directly attributable to residential annual exposures to PM_2.5_ > 15.5 μg/m^3^, below the EU value air quality target value of 25 μg/m^3^. The public health impact of air pollution on physical health is increasingly well understood and studies have shown that improved air quality is associated with a range quantifiable health benefits [52]. In 2016, the World Health Organization (WHO) reported that 91% of the world’s population lives in places where air quality exceeds WHO guideline limits with 4.2 millions of premature deaths being a result of ambient air pollution with 91% of these premature deaths occurring in low- and middle-income countries. Recent evidence also indicated the need for revision of WHO air quality guidelines in even lower limits to protect human health [60]. There should be special attention for innovative measures to improve air quality, such as the Ultra-Low emission Zone in London (ULEZ), the introduction of buses and cars powered by electricity and boldly rethink the way that we plan our car-less visions of cities—an urgency which will be more apparent during the aftermath of the COVID-19 pandemic era [61]. Improving air quality is a tractable, though complex issue [31] and therefore measures to reduce air pollution overall within cities or to reduce individuals’ exposures through behaviour change may represent a potentially impactful primary health measure to mitigate against mental disorders within the urban population.

## Electronic supplementary material

Below is the link to the electronic supplementary material.

Supplementary file1 (DOCX 455 kb)
