# Supplementary material for: Mental health consequences of urban air pollution: prospective population-based longitudinal survey
Source: Soc Psychiatry Psychiatr Epidemiol. 2020 Oct 24;56(9):1587–99. doi: 10.1007/s00127-020-01966-x (PMC7584487; doi:10.1007/s00127-020-01966-x)
Supplement: Supplementary file 1 — Supplementary file1 (DOCX 455 kb) [file 127_2020_1966_MOESM1_ESM.docx]

**Supplementary Online Content**

**Mental health consequences of urban air pollution: prospective population based longitudinal survey**

Ioannis Bakolis^1,2^, Ryan Hammoud ^3^, Robert Stewart^4,5^, Sean Beevers^6^, David Dajnak^6^, Shirlee MacCrimmon^4^, Matthew Broadbent^5^, Megan Pritchard ^5^, Narushige Shiode^7^, Daniela Fecht^8^, John Gulliver^9^, Matthew Hotopf ^4,5^, Stephani L Hatch^5^, Ian S Mudway^6,10^

**Contents**

Materials are presented in the order they are referred to within the main manuscript

|  |  |
| --- | --- |
| 3 | **eFigure S1.** Study area including the London Boroughs of Lambeth and Southwark**.** The study area within the London domain are illustrated in the left-hand panels, with the spatial distribution of NO_2_ illustrated in the upper and lower right-hand panels respectively, based on the average exposures across 2008-2012 at a resolution of 20x20m. |
| 4 | **eTable S1.** Longitudinal associations between air pollutants (NO_2_, NO_x_, O_3_, PM_10_, PM_2.5_) and common mental disorders (CIS-R) physical symptoms (PHQ-15) and self-rated general health (SF-12) with the use of the SELCoH 1 and 2 surveys for non-movers between the two surveys. Odds Ratios (OR) and their corresponding 95% Intervals (CI) represent increase in risk for mental disorders and physical symptoms per IQR increase (μg/m^3^) in air pollutant levels. |
| 5-6 | **eTable S2.** Longitudinal associations between quartile exposure for air pollutants (NO_2_, NO_x_, O_3_, PM_10,_ PM_2.5_) and common mental disorders (CIS-R) and physical symptoms (PHQ-15) and self-rated general health (SF-12) and with the use of the SELCoH 1 and 2 surveys. Odds Ratios (OR) and their corresponding 95% Intervals (CI) represent increase in risk for mental disorders and physical symptoms per quartile (μg/m^3^) increase in air pollutant levels. |
| 7-8 | **eTable S3.** Longitudinal associations between quartile exposure for air pollutants (NO_2_, NO_x_, O_3,_ PM_10,_ PM_2.5_) and common mental disorders (CIS-R) and physical symptoms (PHQ-15) and self-rated general health (SF-12) with the use of the SELCoH 1 and 2 surveys for non-movers. Odds Ratios (OR) and their corresponding 95% Intervals (CI) represent increase in risk for mental disorders and physical symptoms for quartile (μg/m^3^) increase in air pollutant levels. |
| 9 | **eTable S4.** Longitudinal associations between air pollutants (NO_2_, NO_x_, O_3_, PM_10_, PM_2.5_) and and common mental disorders (CIS-R) and physical symptoms (PHQ-15) and self-rated general health (SF-12) with the use of the SELCoH 1 and 2. Odds Ratios (OR) and their corresponding 95% Intervals (CI) represent increase in risk for mental disorders and physical symptoms per IQR increase (μg/m^3^) in air pollutant levels. **Further adjustments for urban noise and air pollutants.** . |
| 10 | **eTable S5.** Longitudinal associations between air pollutants (NO2, NOx, O3, PM10, PM2.5) and common mental disorders (CIS-R) and physical symptoms (PHQ-15) and self-rated general health (SF-12) with the use of the SELCoH 1 and 2 surveys. Odds Ratios (OR) and their corresponding 95% Intervals (CI) represent increase in risk for mental and physical health per IQR annual increase in air pollutant levels *(*μg/m^3^) |
| 11 | **eTable S6.** Longitudinal associations between air pollutants (NO_2_, NO_x_, O_3,_ PM_10,_ PM_2.5_) and common mental disorders (CIS-R) and physical symptoms (PHQ-15) and self-rated general health (SF-12) with the use of the SELCoH 1 and 2 surveys. Odds Ratios (OR) and their corresponding 95% Intervals (CI) represent increase in risk for mental disorders and physical symptoms per IQR increase in air pollutant levels (μg/m^3^). **Further adjustments for perceived neighbourhood deprivation, seasonality and neighbourhood levels of deprivation** |
| 12 | **eTable S7.** Longitudinal associations between air pollutants (NO_2_, NO_x_, O_3,_ PM_10,_ PM_2.5_) and common mental disorders (CIS-R),) with the use of the SELCoH 1 and 2 surveys. Cross-sectional associations between air pollutants (NO_2_, NO_x_, O_3,_ PM_10,_ PM_2.5_) and psychotic experiences with the use of the SELCoH 1 survey. Odds Ratios (OR) and their corresponding 95% Intervals (CI) represent increase in risk mental disorders and physical symptoms per IQR increase in air pollutant levels (μg/m^3^). |
| 13 | **eTable S8.** Longitudinal associations between air pollutants (NO_2_, NO_x_, O_3,_ PM_10,_ PM_2.5_) and anxiety and depression score with the use of the SELCoH 1 and 2 surveys. Odds Ratios (OR) and their corresponding 95% Intervals (CI) represent increase in risk mental disorders and physical symptoms per IQR annual increase in air pollutant levels (μg/m^3^). |
| 14 | **eTable S9.** Effect modification of the longitudinal association of air pollutants (NO_2_, NO_x_, O_3_, PM_10_, PM_2.5_) and common mental disorders (CIS-R) and physical symptoms (PHQ-15) and self-rated general health (SF-12) by individual SES with the use of the SELCoH 1 and 2 surveys. Effect modification of the cross-sectional association of air pollutants (NO_2_, NO_x_, O_3_, PM_10_, PM_2.5_) and psychotic experiences by individual SES with the use of the SELCoH 1 and 2 surveys. |
| 15 | **eTable S10 (multiple imputation with chained equations).** Longitudinal associations between air pollutants (NO_2_, NO_x_, O_3_, PM_10_, PM_2.5_) and common mental disorders (CIS-R) and physical symptoms (PHQ-15) and self-rated general health (SF-12) with the use of the SELCoH 1 and 2 surveys and multiple imputation with chained equations. Odds Ratios (OR) and their corresponding 95% Intervals (CI) represent increase in risk for mental disorders and physical symptoms per IQR increase (μg/m^3^) in air pollutant levels. |

**eFigure S1.** Study area including the London Boroughs of Lambeth and Southwark. The study area within the London domain are illustrated in the left-hand panels, with the spatial distribution of NO_2_ illustrated in the upper and lower right-hand panels respectively, based on the average exposures across 2008-2012 at a resolution of 20x20 metres grid


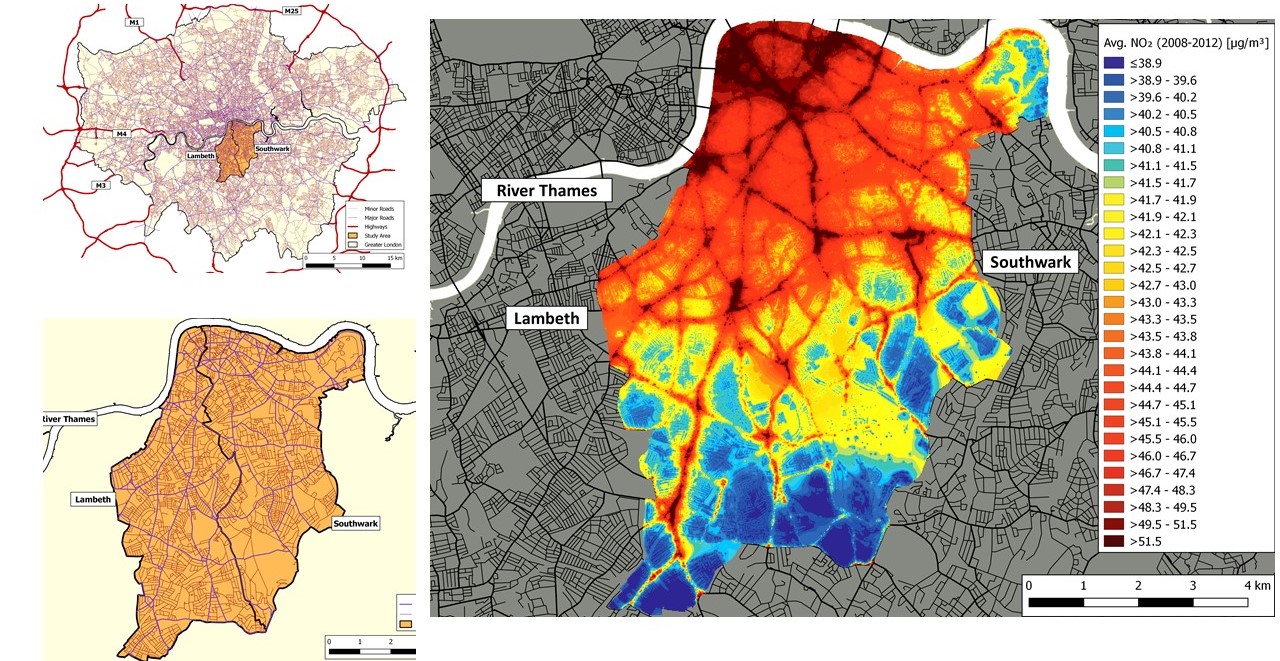


**eTable S1.** Longitudinal associations between air pollutants (NO_2_, NO_x_, O_3_, PM_10_, PM_2.5_) and common mental disorders (CIS-R), physical symptoms (PHQ-15) and self-rated general health (SF-12) with the use of the SELCoH 1 and 2 surveys for non-movers between the two surveys. Odds Ratios (OR) and their corresponding 95% Intervals (CI) represent increase in risk for mental disorders and physical symptoms per IQR increase (μg/m^3^) in air pollutant levels.

|  | **Model 1**^±^ | **Model 2**^±±^ | **Model 3**^±±±^ | **Model 4**^±±±±^ |
| --- | --- | --- | --- | --- |
|  | **OR**  **95% CI** | **OR**  **95% CI** | **OR**  **95% CI** | **OR**  **95% CI** |
| **NO_2_** |  |  |  |  |
| CIS-R | 1.59**  1.13,2.24 | 1.51*  1.08,2.09 | 1.52*  1.10,2.11 | 1.54**  1.12,2.14 |
| PHQ-15 | 1.39*  1.06,1.82 | 1.32*  1.02,1.70 | 1.32*  1.03,1.70 | 1.33*  1.03,1.71 |
| SF-12 | 1.60**  1.24,2.08 | 1.46**  1.14,1.87 | 1.48**  1.15,1.89 | 1.48**  1.16,1.90 |
| **NO_x_** |  | | | |
| CIS-R | 1.54**  1.11,2.12 | 1.47*  1.08,2.00 | 1.48*  1.09,2.01 | 1.50**  1.10,2.03 |
| PHQ-15 | 1.35*  1.05,1.73 | 1.29*  1.02,1.64 | 1.29*  1.02,1.64 | 1.30**  1.03,1.65 |
| SF-12 | 1.52**  1.19,1.94 | 1.40**  1.11,1.76 | 1.42*  1.12,1.79 | 1.42**  1.13,1.79 |
| **O_3_** |  | | | |
| CIS-R | 0.65**  0.47,0.89 | 0.67*  0.49,0.92 | 0.67*  0.49,0.92 | 0.67*  0.49,0.92 |
| PHQ-15 | 0.71**  0.56,0.91 | 0.75*  0.59,0.95 | 0.73**  0.57,0.92 | 0.73**  0.57,0.93 |
| SF-12 | 0.83  0.66,1.05 | 0.87  0.70,1.08 | 0.85  0.68,1.06 | 0.85  0.68,1.06 |
| **PM_10_** |  | | | |
| CIS-R | 1.30  1.00,1.70 | 1.21  0.94,1.55 | 1.21  0.94,1.55 | 1.22  0.95,1.56 |
| PHQ-15 | 1.11  0.89,1.38 | 1.06  0.87,1.30 | 1.05  0.86,1.29 | 1.06  0.86,1.29 |
| SF-12 | 1.21  0.94,1.55 | 1.13  0.94,1.37 | 1.13  0.94,1.37 | 1.13  0.94,1.37 |
| PM_2.5_ |  | | | |
| CIS-R | 1.18*  1.00,1.39 | 1.17  0.99,1.34 | 1.17  0.99,1.35 | 1.18*  1.02,1.37 |
| PHQ-15 | 1.16*  1.02,1.32 | 1.14*  1.01,1.29 | 1.12  0.99,1.27 | 1.12  0.99,1.27 |
| SF-12 | 1.16*  1.02,1.32 | 1.11  0.99,1.26 | 1.11  0.99,1.25 | 1.11  0.99,1.25 |
|  | | | | |

| *0.01<p<0.05 **p<0.01 ^±^Model 1: unadjusted ^±±^Model 2: Adjusted for age, sex, latent classes of SES, smoking status, ethnicity ^±±±^Model 3: Adjusted for age, sex, latent classes of SES, smoking status, ethnicity, frequency of drinking, physical activity^±±±±^Model 4: Adjusted for age, sex, latent classes of SES, smoking status, ethnicity, frequency of drinking, physical activity and L_den_ |
| --- |

**eTable S2.** Longitudinal associations between quartile exposure for air pollutants (NO_2_, NO_x_, O_3_, PM_10,_ PM_2.5_) common mental disorders (CIS-R), physical symptoms (PHQ-15) and self-rated general health (SF-12) and with the use of the SELCoH 1 and 2 surveys. Odds Ratios (OR) and their corresponding 95% Intervals (CI) represent increase in risk mental disorders and physical symptoms per quartile (μg/m^3^) increase in air pollutant levels.

|  |  | **Model 1**^±^ | **Model 2**^±±^ | **Model 3**^±±±^ | **Model 4**^±±±±^ |
| --- | --- | --- | --- | --- | --- |
|  |  | OR  95% CI | OR  95% CI | OR  95% CI | OR  95% CI |
| **NO_2_** |  |  |  |  |  |
| CIS-R | 2 | 1.01  0.63-1.65 | 0.86  0.54-1.36 | 0.85  0.53-1.35 | 0.84  0.53-1.34 |
|  | 3 | 1.27  0.80-2.03 | 1.11  0.71-1.76 | 1.11  0.71-1.76 | 1.10  0.70-1.74 |
|  | 4 | 1.61  0.99-2.61 | 1.41  0.88-2.24 | 1.47  0.93-2.34 | 1.45  0.92-2.31 |
| P for trend |  | 0.015 | 0.033 | 0.023 | 0.017 |
| PHQ-15 | 2 | 0.90  0.60-1.35 | 0.80  0.54-1.16 | 0.82  0.56-1.21 | 0.81  0.55-1.20 |
|  | 3 | 1.34  0.89-2.02 | 1.18  0.79-1.75 | 1.19  0.80-1.78 | 1.18  0.79-1.76 |
|  | 4 | 1.51*  1.00-2.29 | 1.36  0.92-2.02 | 1.44  0.97-2.14 | 1.42  0.95-2.11 |
| P for trend |  | 0.023 | 0.040 | 0.029 | 0.025 |
| SF-12 | 2 | 1.55*  1.08-2.21 | 1.36  0.97-1.90 | 1.35  0.97-1.88 | 1.34  0.96-1.87 |
|  | 3 | 1.64*  1.14-2.36 | 1.43*  1.01-2.03 | 1.45*  1.02-2.05 | 1.43*  1.01-2.03 |
|  | 4 | 1.39  0.95-2.05 | 1.26  0.88-1.81 | 1.33  0.93-1.90 | 1.31  0.92-1.87 |
| P for trend |  | 0.120 | 0.199 | 0.137 | 0.234 |
| NO_x_ |  |  |  |  |  |
| CIS-R | 2 | 0.99  0.61-1.60 | 0.83  0.52-1.32 | 0.81  0.51-1.30 | 0.81  0.50-1.29 |
|  | 3 | 1.13  0.71-1.81 | 1.00  0.64-1.57 | 1.00  0.63-1.57 | 0.98  0.63-1.55 |
|  | 4 | 1.51  0.93-2.46 | 1.31  0.82-2.10 | 1.37  0.86-2.18 | 1.35  0.85-2.15 |
| P for trend |  | 0.017 | 0.035 | 0.024 | 0.019 |
| PHQ-15 | 2 | 0.91  0.61-1.36 | 0.79  0.54-1.16 | 0.82  0.56-1.20 | 0.81  0.55-1.18 |
|  | 3 | 1.31  0.87-1.97 | 1.14  0.77-1.69 | 1.15  0.77-1.72 | 1.13  0.76-1.69 |
|  | 4 | 1.52*  1.01-2.28 | 1.35  0.92-2.00 | 1.43  0.97-2.11 | 1.40  0.95-2.07 |
| P for trend |  | 0.029 | 0.044 | 0.039 | 0.030 |
| SF-12 | 2 | 1.59*  1.11-2.29 | 1.38  0.99-1.93 | 1.37  0.98-1.92 | 1.36  0.97-1.90 |
|  | 3 | 1.54*  1.07-2.20 | 1.39  0.98-1.96 | 1.38  0.98-1.96 | 1.37  0.97-1.93 |
|  | 4 | 1.46  0.99-2.15 | 1.30  0.90-1.86 | 1.36  0.95-1.94 | 1.34  0.94-1.92 |
| P for trend |  | 0.169 | 0.261 | 0.177 | 0.156 |
| O_3_ |  |  |  |  |  |
| CIS-R | 2 | 0.89  0.57-1.38 | 0.91  0.59-1.41 | 0.94  0.61-1.45 | 0.94  0.61-1.46 |
|  | 3 | 0.83  0.51-1.34 | 0.85  0.54-1.35 | 0.87  0.55-1.38 | 0.87  0.55-1.39 |
|  | 4 | 0.64  0.40-1.02 | 0.66  0.41-1.06 | 0.64  0.40-1.02 | 0.64  0.40-1.03 |
| P for trend |  | 0.062 | 0.104 | 0.069 | 0.079 |
| PHQ-15 | 2 | 1.21  0.83-1.77 | 1.14  0.78-1.65 | 1.08  0.74-1.57 | 1.09  0.75-1.59 |
|  | 3 | 1.07  0.71-1.60 | 1.05  0.72-1.54 | 1.04  0.71-1.54 | 1.05  0.71-1.55 |
|  | 4 | 0.79  0.54-1.18 | 0.82  0.56-1.19 | 0.77  0.53-1.12 | 0.77  0.53-1.13 |
| P for trend |  | 0.182 | 0.292 | 0.175 | 0.232 |
| SF-12 | 2 | 0.96  0.68-1.36 | 0.90  0.65-1.26 | 0.86  0.62-1.21 | 0.87  0.62-1.22 |
|  | 3 | 0.84  0.58-1.21 | 0.82  0.57-1.16 | 0.81  0.57-1.15 | 0.81  0.57-1.15 |
|  | 4 | 1.02  0.71-1.45 | 0.99  0.71-1.38 | 0.93  0.67-1.31 | 0.94  0.67-1.31 |
| P for trend |  | 0.706 | 0.754 | 0.491 | 0.453 |
| PM_10_ |  |  |  |  |  |
| CIS-R | 2 | 1.16  0.73-1.85 | 1.15  0.74-1.81 | 1.08  0.69-1.70 | 1.08  0.68-1.70 |
|  | 3 | 0.83  0.53-1.29 | 0.77  0.50-1.19 | 0.77  0.50-1.20 | 0.77  0.50-1.20 |
|  | 4 | 1.91**  1.20-3.05 | 1.71*  1.10-2.66 | 1.65*  1.06-2.56 | 1.64*  1.06-2.54 |
| P for trend |  | 0.040 | 0.090 | 0.091 | 0.099 |
| PHQ-15 | 2 | 0.85  0.58-1.24 | 0.88  0.61-1.27 | 0.86  0.59-1.25 | 0.85  0.59-1.24 |
|  | 3 | 0.68  0.46-1.02 | 0.68*  0.46-1.00 | 0.72  0.49-1.07 | 0.72  0.49-1.06 |
|  | 4 | 1.17  0.80-1.72 | 1.13  0.79-1.62 | 1.13  0.78-1.63 | 1.11  0.77-1.60 |
| P for trend |  | 0.175 | 0.195 | 0.245 | 0.233 |
| SF-12 | 2 | 1.14  0.81-1.59 | 1.12  0.82-1.54 | 1.09  0.80-1.50 | 1.09  0.80-1.49 |
|  | 3 | 1.08  0.78-1.48 | 1.12  0.82-1.53 | 1.23  0.90-1.69 | 1.23  0.90-1.68 |
|  | 4 | 1.08  0.77-1.53 | 1.00  0.72-1.39 | 1.00  0.72-1.39 | 0.99  0.71-1.38 |
| P for trend |  | 0.992 | 0.304 | 0.595 | 0.553 |
| PM_2.5_ |  |  |  |  |  |
| CIS-R | 2 | 1.37  0.85-2.22 | 1.34  0.84-2.14 | 1.33  0.83-2.13 | 1.33  0.83-2.13 |
|  | 3 | 1.38  0.88-2.17 | 1.25  0.80-1.95 | 1.27  0.81-1.98 | 1.26  0.81-1.97 |
|  | 4 | 2.13**  1.32-3.44 | 1.95**  1.23-3.11 | 1.94**  1.22-3.08 | 1.93*  1.22-3.05 |
| P for trend |  | 0.021 | 0.035 | 0.028 | 0.025 |
| PHQ-15 | 2 | 1.22  0.80-1.85 | 1.23  0.82-1.85 | 1.16  0.78-1.75 | 1.16  0.77-1.73 |
|  | 3 | 1.13  0.75-1.70 | 1.11  0.75-1.65 | 1.10  0.74-1.65 | 1.09  0.73-1.62 |
|  | 4 | 1.51*  1.00-2.28 | 1.48*  1.00-2.19 | 1.43  0.97-2.11 | 1.41  0.96-2.09 |
| P for trend |  | <0.001 | <0.001 | <0.001 | <0.001 |
| SF-12 | 2 | 1.12  0.79-1.59 | 1.05  0.76-1.47 | 1.00  0.72-1.38 | 0.99  0.72-1.37 |
|  | 3 | 1.54*  1.08-2.19 | 1.42  1.02-1.97 | 1.45*  1.05-2.01 | 1.44*  1.03-1.99 |
|  | 4 | 1.21  0.83-1.77 | 1.09  0.76-1.56 | 1.06  0.74-1.50 | 1.05  0.74-1.49 |
| P for trend |  | 0.304 | 0.595 | 0.688 | 0.688 |

| *0.01<p<0.05 **p<0.01 ^±^Model 1: unadjusted; ^±±^Model 2: Adjusted for age, sex, latent classes of SES, smoking status, ethnicity ^±±±^Model 3: Adjusted for age, sex, latent classes of SES, smoking status, ethnicity, frequency of drinking, physical activity ^±±±±^Model 4 Adjusted for age, sex, latent classes of SES, smoking status, ethnicity, frequency of drinking, physical activity and L_den_; No_2_ quartile ranges (μg/m3): 24.3–33.4, 33.5–38.4, 38.5.6–51.5, 51.6–82.7; No_x_ quartile ranges (μg/m3): 32.7–52.2, 52.3–64.2, 64.3–100.1, 100.2–184.4; O_3_ quartile ranges (μg/m3): 5.28–24.1, 24.2–32.4, 32.4–42.4, 42.5–55.5; PM_2.5_ quartile ranges (μg/m3): 9.6–12.4, 12.5–14.1, 14.2–15.4, 15.5–23.4; PM_10_ quartile ranges (μg/m3): 13.2–18.6, 18.7–21.5, 21.6–24.3, 24.4–34.1. |
| --- |

**eTable S3.** Longitudinal associations between quartile exposure for air pollutants (NO_2_, NO_x_, O_3,_ PM_10,_ PM_2.5_) and common mental disorders (CIS-R), physical symptoms (PHQ-15) and self-rated general health (SF-12) with the use of the SELCoH 1 and 2 surveys for non-movers. Odds Ratios (OR) and their corresponding 95% Intervals (CI) represent increase in risk for mental disorders and physical symptoms for quartile (μg/m^3^) increase in air pollutant levels.

|  |  | **Model 1**^±^ | **Model 2**^±±^ | **Model 3**^±±±^ | **Model 4**^±±±±^ |
| --- | --- | --- | --- | --- | --- |
|  |  | OR  95% CI | OR  95% CI | OR  95% CI | OR  95% CI |
| NO_2_ |  |  |  |  |  |
| CIS-R | 2 | 1.06  0.61-1.84 | 0.94  0.55-1.51 | 0.92  0.53-1.57 | 0.94  0.55-1.60 |
|  | 3 | 1.44  0.85-2.44 | 1.31  0.78-2.19 | 1.35  0.81-2.25 | 1.39  0.83-2.31 |
|  | 4 | 2.02*  1.14-3.57 | 1.82*  1.05-3.16 | 1.83*  1.06-3.15 | 1.86*  1.08-3.20 |
| P for trend |  | <0.001 | 0.014 | 0.015 | 0.001 |
| PHQ-15 | 2 | 0.89  0.58-1.37 | 0.82  0.54-1.26 | 0.83  0.53-1.29 | 0.84  0.54-1.30 |
|  | 3 | 1.27  0.82-1.97 | 1.16  0.75-1.78 | 1.17  0.76-1.81 | 1.18  0.76-1.83 |
|  | 4 | 1.52  0.97-2.36 | 1.39  0.91-2.12 | 1.41  0.92-2.14 | 1.42  0.93-2.16 |
| P for trend |  | 0.016 | 0.032 | 0.031 | 0.029 |
| SF-12 | 2 | 1.61*  1.05-2.47 | 1.42  0.95-2.12 | 1.38  0.91-2.10 | 1.39  0.92-2.10 |
|  | 3 | 2.08**  1.37-3.17 | 1.84**  1.23-2.75 | 1.91**  1.27-2.87 | 1.93**  1.28-2.89 |
|  | 4 | 2.21**  1.43-3.42 | 1.85**  1.22-2.81 | 1.92**  1.26-2.92 | 1.93**  1.27-2.93 |
| P for trend |  | <0.001 | <0.001 | <0.001 | <0.001 |
| NO_x_ |  |  |  |  |  |
| CIS-R | 2 | 1.20  0.69-2.08 | 1.07  0.63-1.84 | 1.05  0.61-1.80 | 1.07  0.63-1.83 |
|  | 3 | 1.55  0.91-2.64 | 1.41  0.84-2.37 | 1.46  0.87-2.46 | 1.51  0.91-2.52 |
|  | 4 | 2.17**  1.23-3.84 | 1.94*  1.12-3.36 | 1.95*  1.13-3.36 | 1.99*  1.16-3.41 |
| P for trend |  | <0.001 | 0.014 | 0.011 | 0.001 |
| PHQ-15 | 2 | 0.94  0.61-1.46 | 0.88  0.57-1.35 | 0.88  0.57-1.36 | 0.89  0.57-1.38 |
|  | 3 | 1.34  0.86-2.10 | 1.23  0.79-1.90 | 1.24  0.80-1.94 | 1.26  0.81-1.96 |
|  | 4 | 1.58*  1.02-2.47 | 1.44  0.95-2.19 | 1.46  0.96-2.22 | 1.47  0.97-2.24 |
| P for trend |  | 0.019 | 0.035 | 0.032 | 0.030 |
| SF-12 | 2 | 1.51  0.99-2.30 | 1.37  0.92-2.04 | 1.32  0.88-2.00 | 1.33  0.88-2.01 |
|  | 3 | 2.01**  1.31-3.06 | 1.80**  1.20-2.71 | 1.89**  1.25-2.85 | 1.90**  1.26-2.87 |
|  | 4 | 2.21**  1.44-3.40 | 1.86**  1.23-2.81 | 1.93**  1.28-2.92 | 1.94**  1.28-2.93 |
| P for trend |  | <0.001 | <0.001 | <0.001 | <0.001 |
| O_3_ |  |  |  |  |  |
| CIS-R | 2 | 0.96  0.56-1.62 | 0.95  0.58-1.48 | 0.98  0.59-1.62 | 0.98  0.59-1.62 |
|  | 3 | 0.69  0.40-1.17 | 0.68  0.41-1.14 | 0.71  0.42-1.18 | 0.71  0.43-1.17 |
|  | 4 | 0.50*  0.29-0.89 | 0.54*  0.31-0.94 | 0.53*  0.31-0.93 | 0.54*  0.31-0.94 |
| P for trend |  | 0.008 | 0.013 | 0.012 | 0.054 |
| PHQ-15 | 2 | 1.32  0.89-1.96 | 1.30  0.88-1.91 | 1.28  0.87-1.88 | 1.27  0.87-1.87 |
|  | 3 | 0.72  0.46-1.13 | 0.70  0.46-1.09 | 0.73  0.47-1.13 | 0.73  0.47-1.13 |
|  | 4 | 0.64*  0.42-0.98 | 0.72  0.47-1.08 | 0.67  0.44-1.02 | 0.67  0.44-1.02 |
|  |  | <0.001 | 0.017 | <0.001 | <0.001 |
| SF-12 | 2 | 0.93  0.63-1.37 | 0.96  0.65-1.41 | 0.95  0.64-1.42 | 0.95  0.64-1.42 |
|  | 3 | 0.69  0.43-1.09 | 0.74  0.47-1.16 | 0.76  0.48-1.19 | 0.76  0.48-1.19 |
|  | 4 | 0.82  0.54-1.23 | 0.87  0.59-1.29 | 0.83  0.56-1.23 | 0.83  0.56-1.23 |
| P for trend |  | 0.122 | 0.208 | 0.149 | 0.208 |
| PM_10_ |  |  |  |  |  |
| CIS-R | 2 | 0.99  0.60-1.65 | 0.99  0.61-1.61 | 0.92  0.56-1.52 | 0.93  0.56-1.52 |
|  | 3 | 0.86  0.53-1.40 | 0.82  0.51-1.32 | 0.78  0.48-1.25 | 0.77  0.48-1.24 |
|  | 4 | 1.67  0.97-2.86 | 1.41  0.85-2.34 | 1.39  0.84-2.31 | 1.41  0.85-2.33 |
| P for trend |  | 0.054 | 0.141 | 0.133 | 0.145 |
| PHQ-15 | 2 | 1.13  0.71-1.79 | 1.17  0.85-1.83 | 1.07  0.69-1.67 | 1.08  0.69-1.68 |
|  | 3 | 0.80  0.52-1.24 | 0.80  0.52-1.22 | 0.80  0.52-1.23 | 0.80  0.52-1.22 |
|  | 4 | 1.18  0.74-1.87 | 1.07  0.70-1.66 | 1.04  0.68-1.61 | 1.05  0.68-1.61 |
| P for trend |  | 0.357 | 0.571 | 0.617 | 0.745 |
| SF-12 | 2 | 1.07  0.71-1.60 | 0.98  0.67-1.43 | 0.90  0.61-1.33 | 0.90  0.61-1.33 |
|  | 3 | 1.59*  1.09-2.33 | 1.46  1.00-2.15 | 1.49*  1.01-2.22 | 1.49*  1.00-2.21 |
|  | 4 | 1.44  0.97-2.14 | 1.18  0.81-1.73 | 1.17  0.79-1.72 | 1.17  0.80-1.72 |
| P for trend |  | 0.03 | 0.199 | 0.198 | 0.207 |
| PM_2.5_ |  |  |  |  |  |
| CIS-R | 2 | 1.20  0.71-2.03 | 1.23  0.74-2.05 | 1.22  0.72-2.06 | 1.21  0.72-2.05 |
|  | 3 | 1.47  0.90-2.40 | 1.41  0.86-2.29 | 1.45  0.90-2.36 | 1.48  0.91-2.39 |
|  | 4 | 1.90*  1.08-3.34 | 1.72*  1.00-2.96 | 1.75*  1.02-3.01 | 1.77*  1.03-3.05 |
| P for trend |  | 0.048 | 0.076 | 0.061 | 0.076 |
| PHQ-15 | 2 | 1.14  0.70-1.83 | 1.20  0.76-1.91 | 1.08  0.68-1.73 | 1.08  0.68-1.72 |
|  | 3 | 1.22  0.78-1.89 | 1.22  0.80-1.88 | 1.20  0.78-1.85 | 1.21  0.79-1.85 |
|  | 4 | 1.38  0.88-2.17 | 1.32  0.86-2.02 | 1.25  0.82-1.92 | 1.26  0.82-1.94 |
| P for trend |  | 0.026 | 0.039 | 0.073 | 0.056 |
| SF-12 | 2 | 1.33  0.90-1.95 | 1.23  0.84-1.78 | 1.14  0.78-1.66 | 1.14  0.78-1.66 |
|  | 3 | 2.08**  1.39-3.12 | 1.92**  1.30-2.83 | 2.02**  1.36-2.98 | 2.02**  1.37-2.99 |
|  | 4 | 1.76**  1.17-2.66 | 1.54*  1.03-2.31 | 1.50*  1.01-2.24 | 1.51*  1.01-2.25 |
| P for trend |  | 0.025 | 0.077 | 0.083 | 0.098 |

| *0.01<p<0.05 **p<0.01 ^±^Model 1: unadjusted; ^±±^Model 2: Adjusted for age, sex, latent classes of SES, smoking status, ethnicity^±±±^Model 3: Adjusted for age, sex, latent classes of SES, smoking status, ethnicity, frequency of drinking, physical activity ^±±±±^Model 4: Adjusted for age, sex, latent classes of SES, smoking status, ethnicity, frequency of drinking, physical activity and L_den_; No_2_ quartile ranges (μg/m3): 24.3–33.4, 33.5–38.4, 38.5.6–51.5, 51.6–82.7; NO_x_ quartile ranges (μg/m3): 32.7–52.2, 52.3–64.2, 64.3–100.1, 100.2–184.4; O_3_ quartile ranges (μg/m3): 5.28–24.1, 24.2–32.4, 32.4–42.4, 42.5–55.5; PM_2.5_ quartile ranges (μg/m3): 9.6–12.4, 12.5–14.1, 14.2–15.4, 15.5–23.4; PM_10_ quartile ranges (μg/m3): 13.2–18.6, 18.7–21.5, 21.6–24.3, 24.4–34.1 |
| --- |

**eTable S4.** Longitudinal associations between air pollutants (NO_2_, NO_x_, O_3_, PM_10_, PM_2.5_) and common mental disorders (CIS-R), physical symptoms (PHQ-15) and self-rated general health (SF-12) with the use of the SELCoH 1 and 2. Odds Ratios (OR) and their corresponding 95% Intervals (CI) represent increase in risk mental disorders and physical symptoms per IQR increase (μg/m^3^) in air pollutant levels.

|  | **Model**  **1**^±^ | **Model**  **2**^±±^ | **Model**  **3**^±±±^ | **Model**  **4**^±±±±^ | **Model**  **5**^±±±±±^ | **Model 6**^±±±±±±±±^ | **Model**  **7**^±±±±±±±^ |
| --- | --- | --- | --- | --- | --- | --- | --- |
|  | OR  95% CI | OR  95% CI | OR  95% CI | OR  95% CI | OR  95% CI | OR  95% CI | OR  95% CI |
| **NO_2_** |  |  |  |  |  |  |  |
| CIS-R | 1.40* 1.05,1.86 | 1.39*  1.05,1.84 | - | 1.48  0.05,41.88 | 1.31  0.88,1.96 | 1.41  0.94,2.13 | 1.25  0.85,1.84 |
| PHQ-15 | 1.29*  1.02,1.64 | 1.26*  1.01,1.64 | - | 4.71  0.09,25.1 | 1.61  0.98,2.63 | 1.72*  1.02,2.90 | 1.17  0.73,1.86 |
| SF-12 | 1.18  0.95,1.47 | 1.17  0.94,1.46 | - | 37.05  1.06,129.08 | 1.32  0.87,2.03 | 1.66*  1.06,2.60 | 1.42  0.95,2.12 |
| **NO_x_** |  | | | |  |  |  |
| CIS-R | 1.38*  1.05,1.82 | 1.37*  1.04,1.81 | 0.94  0.04,24.98 | - | 1.31  0.88,1.94 | 1.39  0.93,2.07 | 1.24  0.85,1.81 |
| PHQ-15 | 1.28*  1.02,1.61 | 1.25  0.99,1.58 | 0.34  0.01,16.06 | - | 1.58  0.97,2.56 | 1.65  1.00,2.72 | 1.15  0.73,1.80 |
| SF-12 | 1.16  0.93,1.43 | 1.15  0.93,1.42 | 0.04  0.00,1.19 | - | 1.27  0.83,1.94 | 1.54*  1.02,2.36 | 1.34  0.90,1.97 |
| **O_3_** |  | | | |  |  |  |
| CIS-R | 0.78  0.60,1.02 | 0.78  0.60,1.02 | 0.93  0.64,1.34 | 0.93  0.64,1.35 | - | 0.82  0.62,1.08 | 0.82  0.63,1.08 |
| PHQ-15 | 0.86  0.70,1.07 | 0.88  0.71,1.09 | 1.03  0.67,1.59 | 1.03  0.66,1.60 | - | 0.81  0.59,1.12 | 0.84  0.62,1.15 |
| SF-12 | 0.93  0.77,1.13 | 0.93  0.77,1.14 | 1.06  0.73,1.54 | 1.04  0.71,1.52 | - | 0.89  0.67,1.17 | 0.90  0.68,1.19 |
| **PM_10_** |  | | | |  |  |  |
| CIS-R | 1.19  0.97,1.45 | 1.18  0.97,1.44 | 0.99  0.74,1.31 | 0.99  0.75,1.31 | 1.13  0.92,1.39 | - | 0.91  0.61,1.36 |
| PHQ-15 | 1.10  0.93,1.31 | 1.09  0.92,1.29 | 0.92  0.63,1.33 | 0.93  0.65,1.34 | 1.17  0.90,1.51 | - | 0.62  0.38,1.00 |
| SF-12 | 1.00  0.86,1.16 | 1.00  0.85,1.16 | 0.77  0.57,1.05 | 0.80  0.59,1.09 | 0.99  0.79,1.23 | - | 0.86  0.56,1.30 |
| **PM_2.5_** |  | | | |  |  |  |
| CIS-R | 1.18*  1.02,1.37 | 1.18*  1.02,1.37 | 1.09  0.89,1.33 | 1.09  0.89,1.33 | 1.15  0.99,1.34 | 1.25  0.93,1.70 | - |
| PHQ-15 | 1.18**  1.04,1.34 | 1.18**  1.04,1.34 | 1.26  0.97,1.64 | 1.27  0.97,1.64 | 1.31*  1.07,1.59 | 1.83*  1.25,2.67 | - |
| SF-12 | 1.02  0.91,1.15 | 1.02  0.90,1.15 | 0.92  0.73,1.15 | 0.94  0.75,1.17 | 1.03  0.87,1.23 | 1.16  0.83,1.61 | - |

| *0.01<p<0.05 **p<0.01 ^±^Model 1: Adjusted for age, sex, latent classes of SES, smoking status, ethnicity, frequency of drinking, physical activity and **L_night_** ^±±^Model 2: Adjusted for age, sex, latent classes of SES, smoking status, ethnicity, frequency of drinking, physical activity and **L_Aeq,16hr_** ^±±±^Model 3: Adjusted for age, sex, latent classes of SES, smoking status, ethnicity, frequency of drinking, physical activity L_den_ and **NO_2_** ^±±±±^Model 4: Adjusted for age, sex, latent classes of SES, smoking status, ethnicity, frequency of drinking, physical activity and L_den_ and **NOx** ^±±±±±^Model 5: Adjusted for age, sex, latent classes of SES, smoking status, ethnicity, frequency of drinking, physical activity, L_den_ and **O_3_** ^±±±±±±±^ Model 6: Adjusted for age, sex, latent classes of SES, smoking status, ethnicity, frequency of drinking, physical activity, L_den_ and **PM_10_** ^±±±±±±±±^Model 7: Adjusted for age, sex, latent classes of SES, smoking status, ethnicity, frequency of drinking, physical activity, L_den_ and **PM_2.5_** |
| --- |

**eTable S5.** Longitudinal associations between air pollutants (NO_2_, NO_x_, O_3,_ PM_10,_ PM_2.5_) and common mental disorders (CIS-R), physical symptoms (PHQ-15) and self-rated general health (SF-12) with the use of the SELCoH 1 and 2 surveys. Odds Ratios (OR) and their corresponding 95% Intervals (CI) represent increase in risk mental disorders and physical symptoms per IQR annual increase in air pollutant levels (μg/m^3^).

|  | **Model 1**^±^ | **Model 2**^±±^ | **Model 3**^±±±^ | **Model 4**^±±±±^ |
| --- | --- | --- | --- | --- |
|  | OR  95% CI | OR  95% CI | OR  95% CI | OR  95% CI |
| **NO_2_** |  |  |  |  |
| CIS-R | 1.29*  1.04,1.59 | 1.24*  1.01,1.54 | 1.22*  1.01,1.51 | 1.22*  1.01,1.53 |
| PHQ-15 | 0.97  (0.82,1.16) | 0.97  (0.82,1.16) | 0.97  (0.82,1.17) | 0.96  (0.82,1.15) |
| SF-12 | 1.05  0.90,1.23 | 1.04  0.89,1.22 | 1.04  0.89,1.22 | 1.02  0.87,1.20 |
| **NO_x_** |  |  |  |  |
| CIS-R | 1.28*  1.04,1.58 | 1.24*  1.02,1.53 | 1.22*  1.01,1.50 | 1.24*  1.01,1.52 |
| PHQ-15 | 0.96  (0.81,1.14) | 0.96  (0.81,1.14) | 0.97  (0.81,1.15) | 0.95  (0.79,1.13) |
| SF-12 | 1.05  0.90,1.23 | 1.01  0.89,1.12 | 1.01  0.89,1.12 | 1.01  0.87,1.20 |
| **O_3_** |  |  |  |  |
| CIS-R | 0.86  0.65,1.14 | 0.92  0.69,1.22 | 0.95  0.,71,1.28 | 0.95  0.72,1.27 |
| PHQ-15 | 0.85  0.69,1.08 | 0.79  0.72,1.20 | 0.76  0.72,1.17 | 0.76  0.73,1.27 |
| SF-12 | 1.08  0.87,1.34 | 1.07  0.87,1.31 | 1.05  0.84,1.30 | 1.06  0.87,1.20 |
| **PM_10_** |  |  |  |  |
| CIS-R | 1.23  0.93,1.63 | 1.15  0.87,1.53 | 1.12  0.85,1.49 | 1.11  0.83,1.48 |
| PHQ-15 | 1.03  0.84,1.35 | 1.01  0.83,1.31 | 1.01  0.83,1.31 | 1.02  0.83,1.30 |
| SF-12 | 1.08  0.87,1.34 | 1.07  0.87,1.31 | 1.05  0.84,1.30 | 1.06  0.85,1.31 |
| **PM_2.5_** |  |  |  |  |
| CIS-R | 1.13*  1.01,1.42 | 1.15*  1.01,1.43 | 1.18*  1.01,1.48 | 1.17*  1.02,1.47 |
| PHQ-15 | 1.49**  1.23,1.79 | 1.44**  1.20,1.79 | 1.41**  1.17,1.71 | 1.41**  1.16,1.71 |
| SF-12 | 1.02  0.82,1.22 | 1.01  0.80,1.16 | 1.01  0.75,1.15 | 1.03  0.74,1.15 |
|  |  |  |  |  |

| *0.01<p<0.05 **p<0.01 ^±^Model 1: unadjusted ^±±^Model 2: Adjusted for age, sex, latent classes of SES, smoking status, ethnicity ^±±±^Model 3: Adjusted for age, sex, latent classes of SES, smoking status, ethnicity, frequency of drinking, physical activity ^±±±±^Model 4: Adjusted for age, sex, latent classes of SES, smoking status, ethnicity, frequency of drinking, physical activity and L_den_ |
| --- |

**eTable S6.** Longitudinal associations between air pollutants (NO_2_, NO_x_, O_3,_ PM_10,_ PM_2.5_) and common mental disorders (CIS-R), physical symptoms (PHQ-15) and self-rated general health (SF-12) with the use of the SELCoH 1 and 2 surveys. Odds Ratios (OR) and their corresponding 95% Intervals (CI) represent increase in risk mental disorders and physical symptoms per IQR increase in air pollutant levels (μg/m^3^).

|  | **Model 1**^±^ | **Model 2**^±±^ | **Model 3**^±±±^ |
| --- | --- | --- | --- |
|  | OR  95% CI | OR  95% CI | OR  95% CI |
| **NO_2_** |  |  |  |
| CIS-R | 1.36*  1.02,1.81 | 1.44*  1.07,1.92 | 1.37*  1.03,1.83 |
| PHQ-15 | 1.28*  1.00,1.62 | 1.38**  1.09,1.77 | 1.29*  1.02,1.64 |
| SF-12 | 1.17  0.95,1.52 | 1.17  0.94,1.45 | 1.16  0.93,1.42 |
| **NO_x_** |  |  |  |
| CIS-R | 1.35*  1.02,1.79 | 1.42*  1.07,1.89 | 1.36*  1.03,1.81 |
| PHQ-15 | 1.27*  1.00,1.60 | 1.37**  1.08,1.73 | 1.28*  1.02,1.61 |
| SF-12 | 1.15  0.92,1.42 | 1.12  0.90,1.40 | 1.14  0.92,1.42 |
| **O_3_** |  |  |  |
| CIS-R | 0.80  0.61,1.05 | 0.70*  0.52,0.94 | 0.78  0.,60,1.02 |
| PHQ-15 | 0.88  0.71,1.09 | 0.75*  0.59,0.96 | 0.86  0.70,1.07 |
| SF-12 | 0.94  0.77,1.14 | 0.93  0.74,1.16 | 0.94  0.77,1.14 |
| **PM_10_** |  |  |  |
| CIS-R | 1.17  0.96,1.44 | 1.24*  1.00,1.54 | 1.18  0.96,1.44 |
| PHQ-15 | 1.09  0.92,1.30 | 1.20*  1.00,1.44 | 1.28*  1.02,1.61 |
| SF-12 | 0.99  0.85,1.16 | 1.04  0.88,1.23 | 0.99  0.85,1.15 |
| **PM_2.5_** |  |  |  |
| CIS-R | 1.17*  1.01,1.36 | 1.18*  1.01,1.38 | 1.18*  1.01,1.37 |
| PHQ-15 | 1.18**  1.04,1.34 | 1.15*  1.01,1.31 | 1.18**  1.04,1.35 |
| SF-12 | 1.02  0.91,1.15 | 1.04  0.92,1.17 | 1.02  0.91,1.15 |
|  |  |  |  |

| *0.01<p<0.05 **p<0.01 ^±^Model 1: Adjusted for age, sex, latent classes of SES, smoking status, ethnicity, frequency of drinking, physical activity and **perceived neighbourhood disorder** ^±±^Model 2: Adjusted for age, sex, latent classes of SES, smoking status, ethnicity, frequency of drinking, physical activity and **seasonality** ^±±±^Model 3: Adjusted for age, sex, latent classes of SES, smoking status, ethnicity, frequency of drinking, physical activity L_den_ and **index of multiple deprivation** |
| --- |

**eTable S7.** Longitudinal associations between air pollutants (NO_2_, NO_x_, O_3,_ PM_10,_ PM_2.5_) and common mental disorders (CIS-R),) with the use of the SELCoH 1 and 2 surveys. Cross-sectional associations between air pollutants (NO_2_, NO_x_, O_3,_ PM_10,_ PM_2.5_) and psychotic experiences with the use of the SELCoH 1 survey. Odds Ratios (OR) and their corresponding 95% Intervals (CI) represent increase in risk mental disorders and physical symptoms per IQR increase in air pollutant levels (μg/m^3^).

|  | **Model 1**^±^ | **Model 2**^±±^ | **Model 3**^±±±^ | **Model 4**^±±±^ | **Model 5**^±±±^ | **Model 6**^±±±^ | **Model 7**^±±±^ | **Model 8**^±±±^ |
| --- | --- | --- | --- | --- | --- | --- | --- | --- |
|  | OR  95% CI | OR  95% CI | OR  95% CI | OR  95% CI | OR  95% CI | OR  95% CI | OR  95% CI | OR  95% CI |
| **NO_2_** |  |  |  |  |  |  |  |  |
| CIS-R | 1.37*  1.04,1.81 | 1.35*  1.01,1.80 | 1.36*  1.02,1.82 | 1.33*  1.01,1.78 | 1.32*  1.01,1.76 | 1.35*  1.01,1.82 | 1.35*  1.01,1.80 | 1.32*  1.01,1.74 |
| Psychotic experiences | 1.20  0.93,1.54 | 1.19  0.91,1.53 | 1.20  0.93,1.53 | 1.19  0.91,1.52 | 1.19  0.91,1.51 | 1.19  0.92,1.55 | 1.19  0.93,1.53 | 1.18  0.93,1.53 |
| **NO_x_** |  |  |  |  |  |  |  |  |
| CIS-R | 1.36*  1.04,1.79 | 1.34*  1.01,1.78 | 1.35*  1.03,1.82 | 1.33*  1.01,1.74 | 1.31*  1.00,1.71 | 1.34*  1.02,1.78 | 1.34*  1.03,1.78 | 1.31*  1.00,1.71 |
| Psychotic experiences | 1.18  0.92,1.50 | 1.17  0.90,1.52 | 1.17  0.91,1.51 | 1.18  0.91,1.50 | 1.17  0.91,1.51 | 1.17  0.92,1.52 | 1.17  0.92,1.52 | 1.17  0.91,1.51 |
| **O_3_** |  |  |  |  |  |  |  |  |
| CIS-R | 0.80  0.61,1.05 | 0.80  0.60,1.04 | 0.80  0.,60,1.02 | 0.80  0.,60,1.03 | 0.81  0.,61,1.04 | 0.80  0.,62,1.05 | 0.81  0.,61,1.04 | 0.80  0.,62,1.05 |
| Psychotic experiences | 0.73  0.56,0.94 | 0.72  0.56,0.94 | 0.72  0.56,0.94 | 0.73  0.56,0.94 | 0.73  0.56,0.94 | 0.73  0.56,0.94 | 0.72  0.55,0.94 | 0.71  0.56,0.94 |
| **PM_10_** |  |  |  |  |  |  |  |  |
| CIS-R | 1.20  0.99,1.45 | 1.18  0.96,1.44 | 1.18  0.97,1.45 | 1.16  0.95,1.42 | 1.16  0.94,1.42 | 1.17  0.96,1.43 | 1.17  0.94,1.43 | 1.1  0.93,1.44 |
| Psychotic experiences | 1.34*  1.12,1.60 | 1.33*  1.12,1.58 | 1.34*  1.11,1.58 | 1.33*  1.12,1.58 | 1.33*  1.12,1.60 | 1.33*  1.12,1.59 | 1.32*  1.11,1.60 | 1.33*  1.12,1.61 |
| **PM_2.5_** |  |  |  |  |  |  |  |  |
| CIS-R | 1.19*  1.02,1.37 | 1.17*  1.01,1.37 | 1.18*  1.02,1.38 | 1.16*  1.00,1.35 | 1.15*  1.00,1.34 | 1.17*  1.01,1.36 | 1.17*  1.00,1.36 | 1.14*  1.00,1.32 |
| Psychotic experiences | 0.89  0.77,1.03 | 0.88  0.77,1.02 | 0.89  0.77,1.03 | 0.88  0.76,1.02 | 0.88  0.76,1.02 | 0.89  0.77,1.02 | 0.88  0.77,1.02 | 0.87  0.75,1.01 |
|  |  |  |  |  |  |  |  |  |

| *0.01<p<0.05 **p<0.01 ^±^Model 1: Adjusted for age, sex, latent classes of SES, smoking status, ethnicity, frequency of drinking, physical activity and **long-standing illness** ^±±^Model 2: Adjusted for age, sex, latent classes of SES, smoking status, ethnicity, frequency of drinking, physical activity and **asthma** ^±±±^Model 3: Adjusted for age, sex, latent classes of SES, smoking status, ethnicity, frequency of drinking, physical activity L_den_ and **chronic bronchitis** ^±±±^Model 4: Adjusted for age, sex, latent classes of SES, smoking status, ethnicity, frequency of drinking, physical activity L_den_ and **diabetes** ^±±±^Model 5: Adjusted for age, sex, latent classes of SES, smoking status, ethnicity, frequency of drinking, physical activity L_den_ and **high blood pressure** Model 6: Adjusted for age, sex, latent classes of SES, smoking status, ethnicity, frequency of drinking, physical activity L_den_ and **cancer** Model 7: Adjusted for age, sex, latent classes of SES, smoking status, ethnicity, frequency of drinking, physical activity L_den_ and **stroke** Model 8: Adjusted for age, sex, latent classes of SES, smoking status, ethnicity, frequency of drinking, physical activity L_den_ and **previous mental illness** |
| --- |

**eTable S8.** Longitudinal associations between air pollutants (NO_2_, NO_x_, O_3,_ PM_10,_ PM_2.5_) and anxiety and depression score with the use of the SELCoH 1 and 2 surveys. Odds Ratios (OR) and their corresponding 95% Intervals (CI) represent increase in risk mental disorders and physical symptoms per IQR annual increase in air pollutant levels (μg/m^3^).

|  | **Model 1**^±^ | **Model 2**^±±^ | **Model 3**^±±±^ | **Model 4**^±±±±^ |
| --- | --- | --- | --- | --- |
|  | OR  95% CI | OR  95% CI | OR  95% CI | OR  95% CI |
| **NO_2_** |  |  |  |  |
| Anxiety score | 1.06  0.85,1.31 | 1.02  0.82,1.36 | 1.04  0.84,1.30 | 1.04  0.84,1.30 |
| Depression score | 1,25  (1.00,1.57) | 1,16  (0.93,1.57) | 1,18  (0.95,1.46) | 1,17  (0.93,1.45) |
| **NO_x_** |  |  |  |  |
| Anxiety score | 1.05  0.84,1.30 | 1.01  0.81,1.25 | 1.04  0.84,1.28 | 1.03  0.83,1.28 |
| Depression score | 1,25  (1.00,1.56) | 1,18  (0.96,1.47) | 1,19  (0.96,1.46) | 1,17  (0.95,1.45) |
| **O_3_** |  |  |  |  |
| Anxiety score | 0.94  0.76,1.16 | 0.96  0.78,1.19 | 0.93  0.76,1.15 | 0.94  0.76,1.16 |
| Depression score | 0.78  0.63,1.02 | 0.82  0.67,1.01 | 0.80  0.64,1.00 | 0.81  0.66,1.01 |
| **PM_10_** |  |  |  |  |
| Anxiety score | 1.03  0.84,1.15 | 1.02  0.86,1.16 | 1.01  0.81,1.12 | 1.01  0.81,1.11 |
| Depression score | 1,15  (0.98,1.35) | 1.09  (0.93,1.27) | 1.08  (0.93,1.27) | 1.08  (0.92,1.26) |
| **PM_2.5_** |  |  |  |  |
| Anxiety score | 1.01  0.90,1.14 | 1,01  0.88,1.10 | 1,02  0.88,1.11 | 1.02  0.88,1.11 |
| Depression score | 1,10  (0.98,1.24) | 1.07  (0.96,1.20) | 1.07  (0.95,1.20) | 1.06  (0.95,1.19) |

| *0.01<p<0.05 **p<0.01 ^±^Model 1: unadjusted ^±±^Model 2: Adjusted for age, sex, latent classes of SES, smoking status, ethnicity ^±±±^Model 3: Adjusted for age, sex, latent classes of SES, smoking status, ethnicity, frequency of drinking, physical activity ^±±±±^Model 4: Adjusted for age, sex, latent classes of SES, smoking status, ethnicity, frequency of drinking, physical activity and L_den_ |
| --- |

**eTable S9.** Effect modification of the longitudinal association of air pollutants (NO_2_, NO_x_, O_3_, PM_10_, PM_2.5_) and common mental disorders (CIS-R), physical symptoms (PHQ-15) and self-rated general health (SF-12) by individual SES with the use of the SELCoH 1 and 2 surveys. Effect modification of the cross-sectional association of air pollutants (NO_2_, NO_x_, O_3_, PM_10_, PM_2.5_) and psychotic experiences by individual SES with the use of the SELCoH 1 and 2 surveys.

|  | **Model 1**^±^ | **Model 2**^±±^ | **Model 3**^±±±^ | **Model 4**^±±±±^ |
| --- | --- | --- | --- | --- |
|  | OR  95% CI | OR  95% CI | OR  95% CI | OR  95% CI |
| **NO_2_** |  |  |  |  |
| CIS-R | 1.29  0.67,2.50 | 1.29  0.67,2.48 | 1.59  0.72,3.53 | 1.61  0.74,3.50 |
| PHQ-15 | 0.81  0.50,1.29 | 0.76  0.47,1.22 | 0.98  0.54,1.79 | 0.88  0.50,1.51 |
| SF-12 | 1.04  0.67,1.59 | 0.96  0.63,1.47 | 0.88  0.50,1.55 | 0.85  0.51,1.43 |
| Psychotic experiences | 0.95  0.61,1.48 | 1.10  0.70,1.73 | - | - |
| **NO_x_** |  | | | |
| CIS-R | 1.28  0.67,2.45 | 1.26  0.67,2.42 | 1.58  0.72,3.49 | 1.60  0.74,3.43 |
| PHQ-15 | 0.83  0.52,1.31 | 0.77  0.48,1.23 | 1.01  0.56,1.83 | 0.90  0.53,1.51 |
| SF-12 | 1.02  0.67,1.55 | 0.93  0.62,1.42 | 0.87  0.50,1.51 | 0.83  0.51,1.38 |
| Psychotic experiences | 0.98  0.63,1.52 | 1.13  0.72,1.77 | - | - |
| **O_3_** |  | | | |
| CIS-R | 0.91  0.49,1.04 | 0.87  0.48,1.59 | 0.68  0.31,1.47 | 0.64  0.30,1.38 |
| PHQ-15 | 1.17  0.76,1.81 | 1.11  0.71,1.73 | 0.95  0.55,1.64 | 0.98  0.58,1.66 |
| SF-12 | 1.27  0.85,1.88 | 1.29  0.85,1.89 | 1.54  0.92,2.57 | 1.52  0.93,2.48 |
| Psychotic experiences | 0.83  0.52,1.33 | 0.73  0.46,1.15 | - | - |
| **PM_10_** |  | | | |
| CIS-R | 1.04  0.65,1.67 | 1.04  0.64,1.67 | 1.38  0.81,2.36 | 1.36  0.80,2.30 |
| PHQ-15 | 0.79  0.55,1.11 | 0.77  0.55,1.08 | 0.88  0.57,1.35 | 0.90  0.62,1.29 |
| SF-12 | 0.99  0.72,1.37 | 0.96  0.71,1.31 | 0.91  0.61,1.37 | 0.94  0.66,1.35 |
| Psychotic experiences | 0.88  0.64,1.13 | 0.88  0.64,1.21 | - | - |
| **PM_2.5_** |  | | | |
| CIS-R | 1.08  0.76,1.55 | 1.08  0.73,1.50 | 1.17  0.78,1.76 | 1.16  0.78,1.72 |
| PHQ-15 | 0.94  0.72,1.22 | 0.92  0.72,1.19 | 0.95  0.69,1.30 | 0.97  0.74,1.26 |
| SF-12 | 1.17  0.85,1.59 | 1.19  0.93,1.55 | 1.18  0.87,1.60 | 1.19  0.91,1.57 |
| Psychotic experiences | 0.83  0.66,1.04 | 0.83  0.66,1.08 | - | - |

| Model 1: air pollutant (NO_2_, NO_x_, O_3_, PM_10_, PM_2.5_) + latent classes of SES + interaction term of latent classes SES (economically active vs inactive) with air pollutant ^±±^Model 2: air pollutant + latent classes of SES + interaction term of latent classes SES (economically active vs inactive) with air pollutant + age + sex + smoking status + ethnicity + frequency of drinking + physical activity + L_den_ ^±±±^ Model 3: Model 1 for non-movers ^±±±±^ Model 4: Model 2 for non-movers. Note: We had information for psychotic only for SELCOH 1 survey, thus we choose not to present non-movers data. |
| --- |

**eTable S10 (multiple imputation with chained equations).** Longitudinal associations between air pollutants (NO_2_, NO_x_, O_3_, PM_10_, PM_2.5_) and common mental disorders (CIS-R), physical symptoms (PHQ-15) and self-rated general health (SF-12) with the use of the SELCoH 1 and 2 surveys and multiple imputation with chained equations. Odds Ratios (OR) and their corresponding 95% Intervals (CI) represent increase in risk mental disorders and physical symptoms per IQR increase (μg/m^3^) in air pollutant levels.

|  | **Model 1**^±^ | **Model 2**^±±^ |
| --- | --- | --- |
|  | OR  95% CI | OR  95% CI |
| **NO_2_** |  |  |
| CIS-R | 1.38*  1.08,1.77 | 1.50**  1.17,2.12 |
| PHQ-15 | 1.39**  1.10,1.76 | 1.54*  1.11,2.13 |
| SF-12 | 1.18  0.95,1.46 | 1.59**  1.18,2.16 |
| **NO_x_** |  |  |
| CIS-R | 1.35*  1.06,1.72 | 1.43*  1.01,2.01 |
| PHQ-15 | 1.29*  1.03,1.63 | 1.43**  1.04,1.97 |
| SF-12 | 1.19  0.97,1.46 | 1.65**  1.22, 2.24 |
| **O_3_** |  |  |
| CIS-R | 0.81  0.65,1.02 | 0.67*  0.49,0.92 |
| PHQ-15 | 0.83  0.67,1.03 | 0.70**  0.52,0.94 |
| SF-12 | 0.98  0.82,1.19 | 0.82  0.63,1.08 |
| **PM_10_** |  |  |
| CIS-R | 1.19  1.00,1.43 | 1.14  0.90,1.43 |
| PHQ-15 | 1.10  0.95,1.33 | 1.19  0.96,1.48 |
| SF-12 | 0.96  0.83,1.12 | 1.23  1.00,1.51 |
| PM_2.5_ |  |  |
| CIS-R | 1.20*  1.04,1.37 | 1.13*  0.95,1.34 |
| PHQ-15 | 1.23**  1.08,1.40 | 1.24  1.05, 1.47 |
| SF-12 | 1.07  0.95,1.20 | 1.26*  1.07,1.47 |
|  | | |

| *0.01<p<0.05 **p<0.01 ^±^Model 1: Adjusted for age, sex, latent classes of SES, smoking status, ethnicity, frequency of drinking, physical activity, 24-hour average noise ^±±^Model 2 Adjusted for age, sex, latent classes of SES, smoking status, ethnicity, frequency of drinking, physical activity and L_den_ for non-movers |
| --- |
